# Disruption of podocyte cytoskeletal biomechanics by dasatinib leads to nephrotoxicity

**DOI:** 10.1038/s41467-019-09936-x

**Published:** 2019-05-03

**Authors:** Rhodora C. Calizo, Smiti Bhattacharya, J. G. Coen van Hasselt, Chengguo Wei, Jenny S. Wong, Robert J. Wiener, Xuhua Ge, Nicholas J. Wong, Jia-Jye Lee, Christina M. Cuttitta, Gomathi Jayaraman, Vivienne H. Au, William Janssen, Tong Liu, Hong Li, Fadi Salem, Edgar A. Jaimes, Barbara Murphy, Kirk N. Campbell, Evren U. Azeloglu

**Affiliations:** 1 Division of Nephrology, Department of Medicine, Icahn School of Medicine at Mount Sinai, New York, NY 10029 USA; 2 Department of Pharmacological Sciences, Icahn School of Medicine at Mount Sinai, New York, NY 10029 USA; 3 Department of Mechanical Engineering, Columbia University, New York, NY 10027 USA; 4 Department of Neuroscience, Icahn School of Medicine at Mount Sinai, New York, NY 10029 USA; 5 Department of Microbiology, Biochemistry and Molecular Genetics, Rutgers University–New Jersey Medical School, Newark, NJ 07103 USA; 6 Department of Pathology, Icahn School of Medicine at Mount Sinai, New York, NY 10029 USA; 7 Renal Service, Memorial Sloan Kettering Cancer Center, New York, NY 10065 USA

**Keywords:** Actin, Drug safety, Podocytes

## Abstract

Nephrotoxicity is a critical adverse event that leads to discontinuation of kinase inhibitor (KI) treatment. Here we show, through meta-analyses of FDA Adverse Event Reporting System, that dasatinib is associated with high risk for glomerular toxicity that is uncoupled from hypertension, suggesting a direct link between dasatinib and podocytes. We further investigate the cellular effects of dasatinib and other comparable KIs with varying risks of nephrotoxicity. Dasatinib treated podocytes show significant changes in focal adhesions, actin cytoskeleton, and morphology that are not observed with other KIs. We use phosphoproteomics and kinome profiling to identify the molecular mechanisms of dasatinib-induced injury to the actin cytoskeleton, and atomic force microscopy to quantify impairment to cellular biomechanics. Furthermore, chronic administration of dasatinib in mice causes reversible glomerular dysfunction, loss of stress fibers, and foot process effacement. We conclude that dasatinib induces nephrotoxicity through altered podocyte actin cytoskeleton, leading to injurious cellular biomechanics.

## Abstract (web summary)

Kinase inhibitors used in chemotherapy are known for their adverse effects on kidney physiology. Here, Calizo et al. show that dasatinib is associated with a higher risk of glomerular toxicity compared to other kinase inhibitors, due to deleterious effects on cytoskeletal biomechanics in podocytes.

## Introduction

Toxicity of targeted therapeutics, such as protein kinase inhibitors (KIs), is a growing concern in clinical oncology as many of these new agents are used as long as clinical benefits are being observed^1^. This is particularly important for chronic kidney disease (CKD) patients since loss of renal function has been associated with increased prevalence of cancer^2^, whereby nephrotoxicity could effectively render targeted treatments inaccessible to a particularly susceptible group. Accordingly, understanding the mechanisms of toxicity of targeted therapies can be of great utility in the design of improved drug dosage protocols, early identification of adverse reactions (ADR), and preliminary screening of patients that may be susceptible to nephrotoxicity. Several targeted therapeutics, such as KIs against VEGF receptor, BRAF, PDL, RANKL, and mTOR (list of all protein names and acronyms are listed in Supplementary Table 1) have been shown to have nephrotoxic effects^3^; however, mechanisms of nephrotoxicity vary and most of them are not clearly understood. For example, patients on multi-kinase inhibitors that target VEGF receptor have increased risk of developing hypertension along with nephrotic-range proteinuria possibly as a secondary complication^4^.

Downing et al. recently published meta-analyses showing that despite years-long clinical trials, 32% of all new FDA-approved drugs had post-market safety events, resulting in withdrawals and black-box warnings within six years of their approval^5^. This study showed that both preclinical studies and multiphasic clinical trials could fail to identify serious ADR of new drugs, in particular those effects that could develop over long periods of time, such as CKD. This is particularly critical in the case of nephrotoxicity because even subclinical acute injuries could increase the risk for developing CKD and other non-renal morbidities later in life^6^.

In the current study, we therefore analyze patient ADR reports, including post-marketing data, to quantify the relative risk for nephrotoxicity in cancer patients that received treatment with KIs. We show that BCR-ABL1 inhibitor dasatinib used for chronic myeloid leukemia (CML), has the highest reporting odds ratio for nephrotoxic ADR with no apparent increase of hypertension risk. Using an integrative approach that includes high-throughput imaging, single-cell analytics, and phosphoproteomics, we identify the cellular, molecular, and biophysical characteristics of dasatinib-associated nephrotoxicity and further highlight the potential mechanisms that lead to podocyte pathophysiology and glomerular dysfunction both in vitro and in vivo. Our results comprise quantitative characterization of dasatinib-induced nephrotoxicity, linking molecular processes and the associated biophysical changes with clinical phenotype. They further provide a framework for future studies to identify off-target renal effects and offer a mechanism that can be used in early drug development and safety studies. We also identify a non-hypertension inducing nephrotoxic KI that leads to glomerular damage instead of acute tubular injury. Additionally, we show that perturbations with even mild cytoskeletal effects may have a substantial impact on glomerular physiology, further highlighting a previously overlooked relationship between multiple subthreshold acute injuries and development of CKD later in life.

## Results

### Dasatinib associates with renal injury but not hypertension

**Fig. 1:**
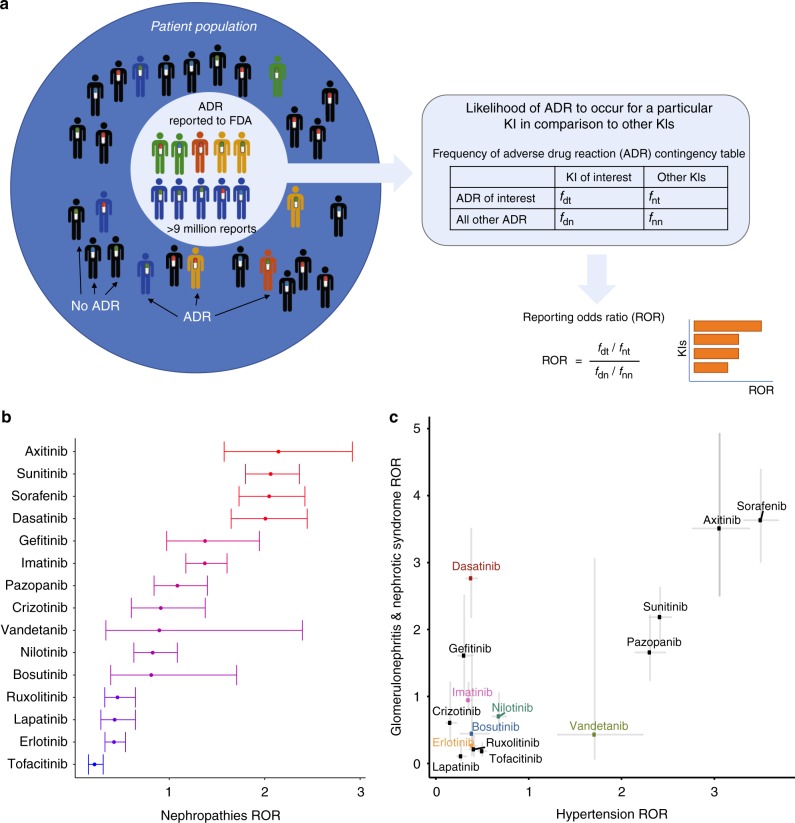
Mining of the FDA Adverse Reporting System (FAERS) database demonstrates that dasatinib is associated with high risk for nephrotoxicity. **a** Workflow for FAERS database mining. **b** Dasatinib ranked as one of the highest reporting odds ratio (ROR) for all nephropathies among other KIs in the FAERS database. **c** When the KI ROR values for glomerular specific nephropathies are plotted against those for hypertension, dasatinib is seen as a clear outlier with high risk for glomerular disorder and a relatively low risk for hypertension

We previously reported a crowd-sourcing approach to rank drug associated ADR in the FDA Adverse Event Reporting System (FAERS) database^7^. Our data mining approach (Fig. 1a) showed that various KIs exhibit significantly increased reporting odds ratios (RORs) for nephrotoxic ADR, which have been unobserved or under-reported before. Along with VEGF receptor inhibitors axitinib, sorafenib and sunitinib, dasatinib showed a high risk for nephropathies compared to other KIs (Fig. 1b). When we further dissected subcategories of ADR, we noted that while dasatinib had one of the highest RORs for glomerular events, it ranked among the lowest for tubular events (Supplementary Fig. 1). Dasatinib is a multi-kinase inhibitor targeting ABL1, SRC, and LCK^8^. While there was a number of other KIs that had similar target profiles (such as bosutinib and nilotinib), none of these ranked as high as dasatinib, suggesting that another off-target kinase (or a combination) was responsible for the renal ADR. Previously, a few case reports have noted nephrotic-range proteinuria on dasatinib patients that was attributed to its inhibitory effect on VEGF receptor^9^ as VEGF receptor inhibitors have been shown to induce renal ADR associated with vascular toxicity and hypertension^10^ with secondary renal dysfunction^4^. To test the connection between dasatinib-induced hypertension and nephrotic syndrome, we determined the correlation between RORs for hypertension and nephrotoxicity for FDA-approved KIs. As expected, the two risk factors for most of the KIs showed a strong correlation, where an increased ROR for hypertension was accompanied by an increased ROR for glomerular injury (Fig. 1c). We note that dasatinib did not cluster with any of the VEGF receptor inhibitors that showed high risk for hypertension. In fact, it was completely outside the observed correlative relationship between the two risk factors with one of the highest observed odds ratio for nephrotic syndrome and a relatively low risk for hypertension. This finding suggested that the nephrotoxicity of dasatinib was primarily through its effect on glomerular podocytes and independent of systemic or glomerular VEGF inhibition.

### Dasatinib disrupts actin cytoskeleton and FA architecture

To experimentally test the hypothesis that the nephrotoxic outcome for dasatinib was through its effect on podocytes, we performed immunofluorescence-based high-content image analysis (HCA) coupled with quantitative single-cell morphometry on podocytes treated with KIs that represented varying ROR scores from the data-mining results (Supplementary Fig. 1). We hypothesized that among the three constituent cell types of the glomerular filtration barrier, (i.e., endothelial cells, mesangial cells, and podocytes), KIs would most significantly impact podocytes because of their highly elaborate and specialized cytoskeletal architecture. Differentiated immortalized mouse podocytes, which have been shown to recapitulate podocyte injury in vivo^11^, were treated with KIs representative of various targets: BCR-ABL1 inhibitors dasatinib (DAS), imatinib (IMA), nilotinib (NIL), bosutinib (BOS), the VEGF receptor inhibitor vandetanib (VAN), and EGF receptor inhibitor erlotinib (ERL). Key metrics and pharmacological information for the selected drugs are summarized in Supplementary Table 2.

**Fig. 2:**
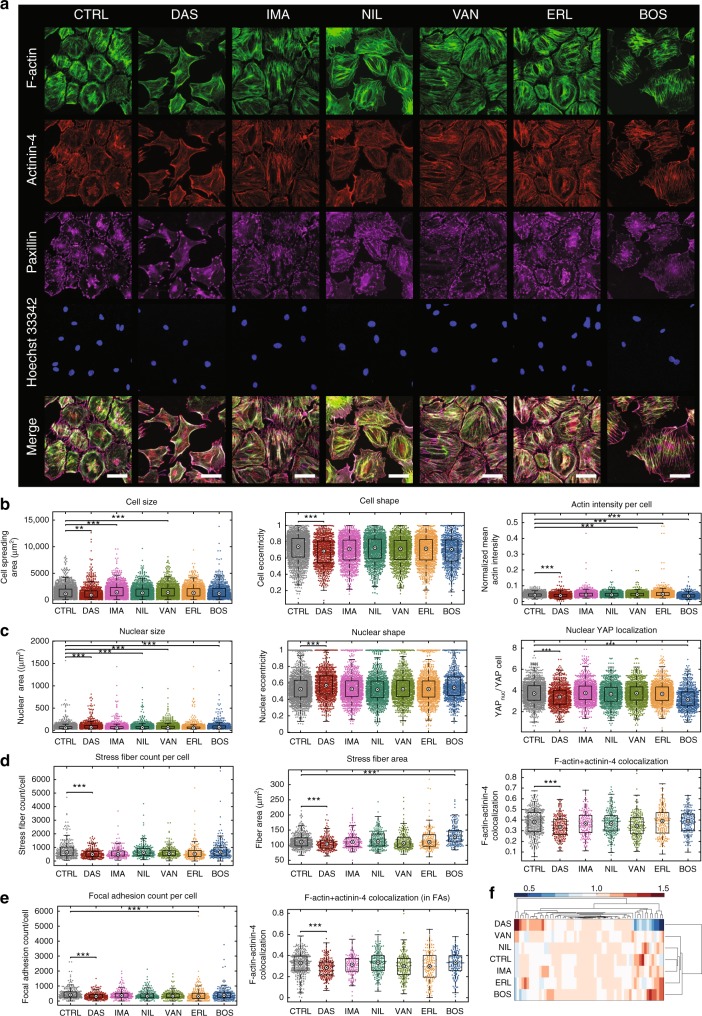
Podocyte morphometrics show that focal adhesion architecture and stress fiber formation are severely impacted by dasatinib. **a** Conditionally immortalized differentiated podocytes treated for one-hour with 2 µM kinase inhibitor were stained for F-actin (phalloidin), α-actinin-4, paxillin, and nuclei (Hoechst 33342) to assess cytoskeletal and focal adhesion architecture (scale bars = 50 µm). Scatter plots of **b** cell size, shape, and average actin intensity; **c** nuclear size, shape, and nuclear YAP localization; **d** stress fiber count per cell, stress fiber area, and F-actin + actinin-4 colocalization; **e** and focal adhesion number per cell and F-actin + actinin-4 colocalization at the focal adhesion sites in podocytes treated with KIs for 24 h. Central bullseye within the distributions highlight population medians, boxes highlight the middle quartiles. Sample sizes are *n*_CTRL_ = 2313; *n*_DAS_ = 491; *n*_IMA_ = 1011; *n*_NIL_ = 956; *n*_VAN_ = 788; *n*_ERL_ = 867; and *n*_BOS_ = 749 for cellular and nuclear plots, which were captured at 200× magnification. Focal adhesion and stress fiber analysis was performed with images captured at 400× magnification, which had the following sample sizes: *n*_CTRL_ = 425; *n*_DAS_ = 221; *n*_IMA_ = 183; *n*_NIL_ = 231; *n*_VAN_ = 220; *n*_ERL_ = 227; and *n*_BOS_ = 196. All experimental groups were taken from minimum of three wells of a 96-well plate (***p < 0.001, Kruskal–Wallis one-way ANOVA followed by Tukey post-hoc multiple comparison). **f** Heatmap representation of all of the analyzed morphometric parameters (normalized median value plotted; *n* = 60) showing that dasatinib is strongly separated from all the other KIs in terms of its effect on cellular, cytoskeletal and nuclear morphology

**Fig. 3:**
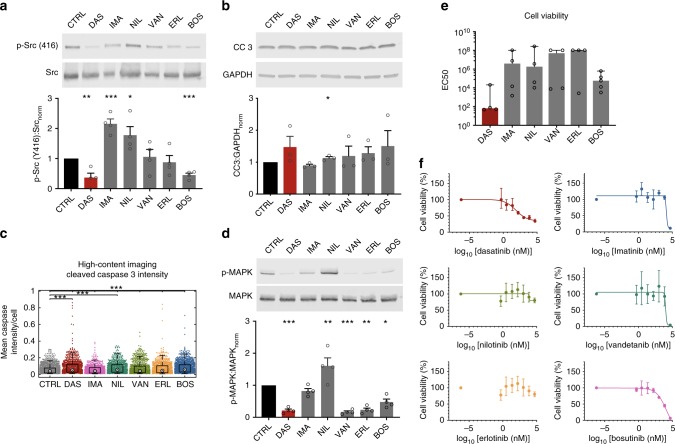
Dasatinib does not have a unique effect on cell survival compared to other KIs. **a** Western blots for p-Src (Y416) show that both bosutinib and dasatinib at 2 µM strongly inhibit SRC (mean ± SEM, *p < 0.05, unpaired *t*-test). **b** Western blot (mean ± SEM; *p < 0.05, unpaired t-test) and **c** immunofluorescence-based high-content image analysis (median ± middle quartiles; ***p < 0.001, Kruskal-Wallis one-way ANOVA followed by Tukey post-hoc multiple comparison) both showed small increase in apoptosis, as quantified by levels of cleaved caspase 3 (CC 3); however there were no significant differences between dasatinib and other KIs. **d** Western blots for p-MAPK, a key kinase for cell survival, showed that dasatinib is not unique in its ability to block this pathway (mean ± SEM; *p < 0.05, unpaired *t*-test). **e** Dasatinib had a major effect on podocyte viability after 24 h of treatment, as assessed by four-parameter half-maximal effective concentration (EC50) fit of the MTT dose-response curve. Even though dasatinib had the lowest EC50, it was not significantly different from other KIs (geometric mean ± geometric SD, *p* = 0.169, Kruskal–Wallis one-way ANOVA). **f** Representative cell survival curves showing percent viability normalized to vehicle control

Shown in Fig. 2a are representative images of podocytes that were treated with 2 µM KIs for 1-h and stained with phalloidin, anti-actinin-4 and anti-paxillin antibodies, and Hoechst 33342 to visualize F-Actin, crosslinked podocyte-specific stress fibers, focal adhesions (FA), and nuclei, respectively. Qualitatively, podocytes treated with dasatinib showed a marked decrease in cell-to-cell contacts, cell size and number of FA. To quantitatively determine the effect of KI treatment on podocyte phenotype, we quantified podocyte cell and nuclear morphology, actin fiber organization and FA characteristics using high-content image analysis (HCA; Supplementary Fig. 2). Over 60 nuclear and cell features were extracted from cells stained for nuclear, focal adhesion and cytoskeletal markers; features included cellular and nuclear size, aspect ratio, stress fiber characteristics, and FA size and number. Compared to control, dasatinib treated cells had significantly smaller cell spreading area (Fig. 2b), with the highest decrease observed among all other KIs (three replicates, ***p < 0.001, nonparametric Kruskal-wallis one-way ANOVA, followed by Tukey post-hoc multiple comparison against control). There was also significant reduction in normalized mean actin intensity for dasatinib treated cells, while erlotinib had a significant increase suggesting an enhancement of cytoskeletal stability by EGF receptor inhibition, as noted by others^12,13^. A similar effect was observed in nuclear size and shape and YAP nuclear localization (Fig. 2c). Number and size of actin stress fibers and colocalization of α-actinin-4 with F-actin (Fig. 2d) decreased significantly only in the dasatinib group, concordant with the observed actin cytoskeletal and FA disruption. Dasatinib treated podocytes also showed significantly lower number of FA per cell compared to control (Fig. 2e), with concomitant decrease in colocalization of actin-crosslinker α-actinin-4. Surprisingly, other BCR-ABL1 inhibitors, including first generation inhibitor imatinib, as well as VEGF receptor inhibitor vandetanib whose downstream effector SRC could affect FA, did not markedly impact the cytoskeletal and FA integrity of podocytes. When the median values for all of the quantified cellular, nuclear, cytoskeletal, and focal adhesion morphometric parameters for different conditions are compared using unsupervised clustering with Euclidean distance, dasatinib showed a clear separation from all other conditions (Fig. 2f). This suggests that the cytoskeletal disruption mediated by dasatinib may not be attributed to its inhibition of BCR-ABL1 activity, but may be due to off-target actin-stabilizing kinases. Furthermore, failure of vandetanib to phenocopy the actin-disrupted phenotype of dasatinib treated podocytes suggests that nephrotic syndrome induced by dasatinib is unlikely to stem from VEGF receptor inhibition. Lastly, bosutinib, a potent SRC inhibitor, did not disrupt cytoskeletal and FA integrity of podocytes suggesting that SRC inhibition was not sufficient to phenocopy the cytoskeletal disruption observed under dasatinib, although causing similar decrease in cell viability (Supplementary Fig. 3). We confirmed that SRC activity was selectively inhibited by both bosutinib and dasatinib via western blots (Fig. 3a).

We further performed expansive timecourse studies to quantify the cellular and nuclear morphometric remodeling following 30 min to 48 h of drug treatment with all the KIs at both 100 nM and 2 µM concentrations; the most important morphometric features are shown in Supplementary Fig. 4-7. We note that the effects of dasatinib on cell shape, actin stress fiber formation, and YAP localization were all observed at the clinically relevant lower concentration of 100 nM (Supplementary Fig. 6-7). Even though dasatinib’s effect was greatest, significant perturbations in cell and nuclear size and shape were observed in most KI-treated cells compared to the control (vehicle-treated) cells within 1 h of drug treatment. Interestingly, some of the significant morphological changes induced by the KIs disappeared after 48 h, suggesting that the changes observed after one-hour signify acute short-term effects, as cells are perturbed from their initial cellular states. After 48 h, lasting and robust changes in cell and nuclear size and more critically significant alterations to the cytoskeleton were observed almost exclusively in the dasatinib group. The unique impact of dasatinib on cellular morphology was also clearly visualized when the 60 morphometric parameters from all KI conditions were clustered using the t-distributed stochastic neighbor embedding (tSNE) method (Supplementary Fig. 8). HCA findings were also recapitulated with immortalized human podocytes, which revealed the same phenotype (actin-disruption and decreased cell size) when treated with dasatinib, as compared to other KIs albeit with increased variability (Supplementary Fig. 9).

### Reduced viability and apoptosis follow cytoskeletal damage

Another potential explanation for the increased risk of glomerular toxicity with dasatinib could be reduced viability and loss of podocytes. To assess whether dasatinib uniquely impacted cell survival and apoptosis in podocytes, we compared cleaved caspase 3 (CC3) expression across different KIs via western blotting. We found that dasatinib marginally increased apoptosis in podocytes similar to the mild apoptosis observed with other tested KIs (Fig. 3b). We further confirmed this finding using two other methods: quantitative high-content image analysis with CC3 immunofluorescence staining (Fig. 3c) and flow cytometry-based Annexin V assay (Supplementary Fig. 10). While all techniques similarly suggested minimal increase in apoptosis, there were some differences in the exact amount most likely due to differential sensitivity of the assays and the timing of apoptosis they measure. Regardless, none of the assays detected increased apoptosis uniquely in the dasatinib group. Furthermore, another key pathway necessary for survival, MAPK signaling, was not uniquely affected by dasatinib either (Fig. 3d). While dasatinib decreased p-MAPK levels as expected, MAPK phosphorylation was not impacted solely by dasatinib but also by VEGF and EGF receptor inhibitors vandetanib and erlotinib. Taken together, these results suggested that the cytoskeletal disruption observed in dasatinib samples is not linked to increased apoptosis.

**Fig. 4:**
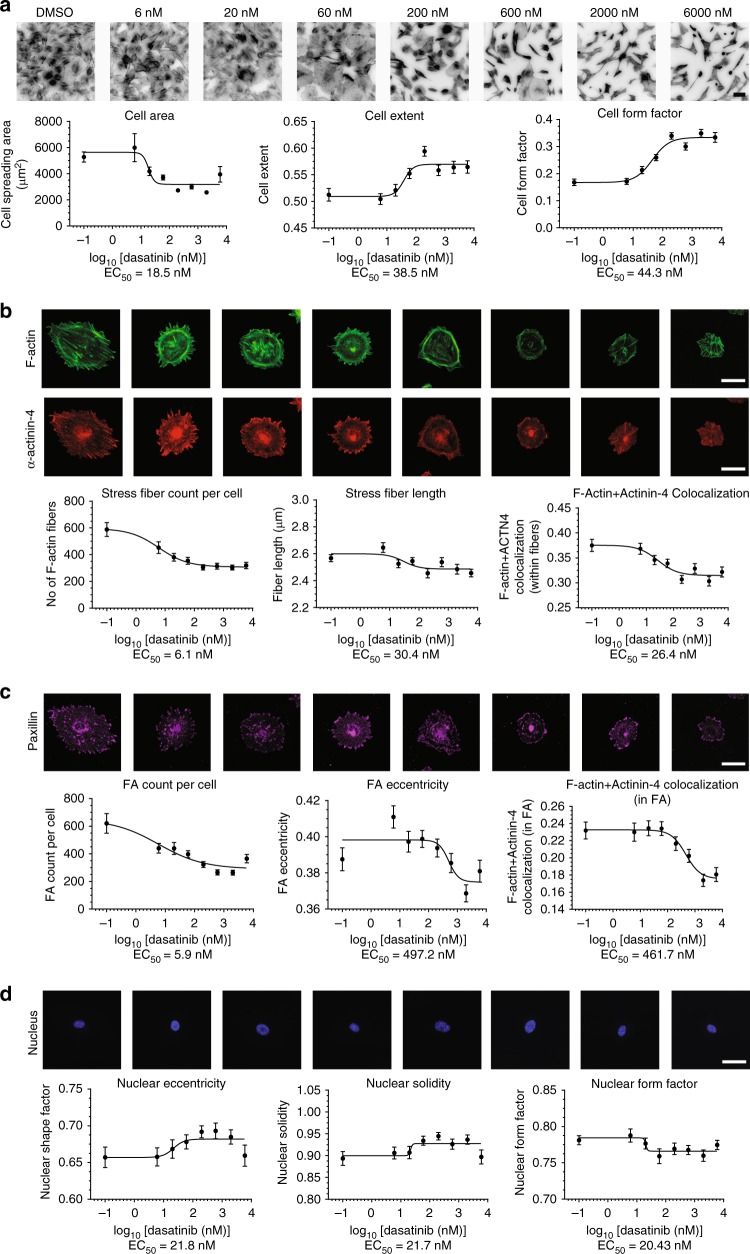
Cytoskeletal effects of dasatinib occur at lower concentrations than those observed clinically. **a** Dose-response for dasatinib with respect to cell morphology was evaluated using high-content image analysis to quantify cell spreading area, extent and form factor. Top: podocytes at varying concentrations of dasatinib stained for F-actin, inverted grayscale lookup table (scale bar = 100 µm). **b** Cytoskeletal effects of dasatinib were quantified by segmenting high magnification (400×) images of F-actin and α-actinin-4, which showed significant effects at 6.1 nM for total number of stress fibers. **c** Number of focal adhesions were significantly reduced at 5.9 nM as quantified by segmentation of paxillin immunofluorescence images at 400×. **d** Changes in nuclear morphology mirrored changes in cell spreading area and were significantly altered at approximately 20 nM of dasatinib. Scale bars, **b**–**d** = 50 µm

To quantitatively determine the comparative effect of dasatinib on viability, we performed the colorimetric MTT (3-(4, 5-dimethylthiazolyl-2)-2, 5-diphenyltetrazolium bromide) viability assay (*n* = 4). Accordingly, dasatinib had a half-maximal effective concentration (EC50) of 253 ± 19 nM (geometric mean ± geometric SD) on cultured podocytes (Fig. 3e, f). While this was not a particularly lethal value, we note that it was still the lowest among all the tested inhibitors, albeit not statistically significant compared to other KIs (*p* = 0.169, nonparametric Kruskal–Wallis test). We then performed a dose-escalating HCA assay to evaluate the changes in morphological, cytoskeletal and focal adhesion parameters at different concentrations of dasatinib (Fig. 4). Compared to the effective kill concentration, dasatinib had a much lower half-maximal effective concentration for inducing morphological and cytoskeletal changes. EC50 values for cell shape and spreading area metrics were between 18.5 and 44.3 nM (Fig. 4a). We observed that of all the cellular parameters quantified in our HCA assay, the highest sensitivity to dasatinib was with stress fiber and FA density. In particular, number of F-actin stress fibers and FA were significantly reduced at EC50 of 6.1 and 5.9 nM (Fig. 4b, c), respectively, while the EC50 values for nuclear architecture were between 20.4 and 21.8 nM (Fig. 4d). Considering clinically measured maximal plasma concentrations of 130 nM^14^, our results clearly demonstrate that cytoskeleton disruption, which occurs at much lower concentrations, precedes potential loss of cell viability, and it is much more likely to be responsible for the clinical phenotype.

### Dasatinib targets actin-related pathways

**Fig. 5:**
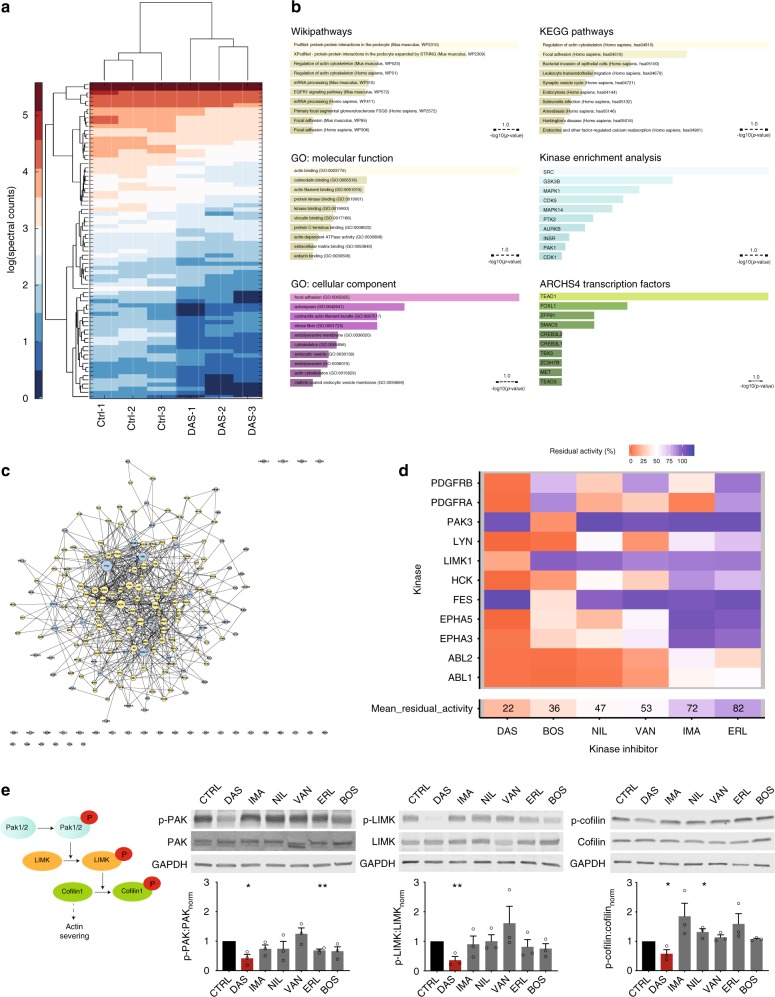
Phosphoproteomics reveal that processes associated with the actin cytoskeleton are specifically perturbed by dasatinib. **a** Heatmap for spectral counts of differentially expressed phospho-tyrosine enriched proteins as quantified by LC-MS/MS. Complete list of proteins plotted in the heatmap are shown in Supplementary Table 3. **b** Enrichment analyses using Wikipathways, KEGG Pathways and other ontology libraries coherently associate focal adhesions and the actin cytoskeleton with the differential phosphoproteome. Highest transcriptional enrichment was for TEAD1 suggesting potential inhibition of the Hippo pathway. **c** Predicted protein-protein interaction network using the differentially phosphorylated proteins as seed nodes (cyan) and one intermediate neighbor (yellow) is highly interconnected. Node size signifies connectivity. Complete list of network associated proteins and number of connections are listed in Supplementary Table 4. **d** KINOMEscan analysis reveals that the activity of actin-associated kinases are disproportionately impacted by dasatinib compared to all other tested KIs; in particular LIM kinase (LIMK1) is identified as a kinase that is inhibited exclusively by dasatinib. **e** Active phosphorylation of kinases from the Rac/Cdc42 pathway (PAK1/2, LIMK1, and cofilin), after treatment with 2 µM KIs for 1 h, show strong, exclusive and statistically significant inhibitory effect for dasatinib but none of the other tested KIs

To have a comprehensive understanding of the systems-level impact of dasatinib, we performed label-free quantitative proteomic analysis that compares the phosphoproteomes of untreated and dasatinib-treated podocytes. Podocytes were lysed and prepared for liquid chromatography followed by tandem mass spectrometry (LC-MS/MS) following enrichment of tyrosine-phosphorylated proteins using immunoprecipitation as previously described^15^. Briefly, cultured podocytes were treated with dasatinib or vehicle-control for one-hour and then lysed in immunoprecipitation (IP) lysis buffer. Tyrosine phosphorylated proteins were pulled down overnight at 4 °C using anti-mouse ferromagnetic beads that were pre-coated with mouse anti-phospho-tyrosine primary antibodies. LC-MS/MS analysis was then used to identify the proteins in IP lysates and quantify their relative abundances based on label-free spectral counting^16^. This process resulted in identification of 2130 distinct phosphoproteins. Only proteins with at least two peptides from both dasatinib and control samples were compared using a multiple hypothesis corrected *t*-test (Fig. 5a). Accordingly, the most significantly impacted phosphoprotein was the FA protein paxillin (18.7 ± 1.2 vs. 3.3 ± 0.6 unique spectra in CTRL vs. DAS, mean ± SEM, *p* = 3.3.10^−5^), which was independently recapitulated via subsequent western blotting.

The majority of 76 statistically significant downregulated proteins were related to regulation of the actin cytoskeleton and FA (Supplementary Table 3). We used enrichment analysis^17^ to identify which signaling pathways and ontology terms were associated with the phosphoproteins inhibited by dasatinib treatment in podocytes (Fig. 5b). The ontology term “Protein-protein interactions in the podocyte” from WikiPathways reached the highest significance (*p* = 1.14 × 10^-8^) consistent with our hypothesis that the foot processes (FP) and slit diaphragm architecture in podocytes are impacted by the altered signaling landscape. The next highest associations were with “Regulation of actin cytoskeleton” (*p* = 7.07 × 10^−7^) and “Focal adhesion” (*p* = 4.2 × 10^−5^) from KEGG pathways, highlighting the prominent role of actin-mediated signaling during dasatinib treatment. Gene ontology enrichment showed similar findings with “focal adhesion”, “stress fiber”, and “actin cytoskeleton” ranking as the highest cellular components. We then used network analysis whereby intermediate genes from the human interactome were combined with the 76 significantly downregulated phosphoproteins to determine the protein–protein interaction network that is targeted by dasatinib. Our analysis revealed a single interconnected island with 190 nodes and 741 edges that was also highly focused on actin regulators and FA/integrin signaling (Fig. 5c; complete list of nodes are listed in Supplementary Table 4). In order to test the specificity of this network, we used Monte Carlo simulations to create background networks from random 76 proteins identified among the original 2130 LC-MS/MS targets. When compared to these background networks, dasatinib network was the largest and most interconnected one based on all measured metrics (Supplementary Fig. 11), confirming that the phosphoproteomic assay has correctly identified the actin-centric signaling foci impacted by dasatinib.

### LIM kinase pathway is uniquely impacted by dasatinib

While quantitative phosphoproteomics provided additional confirmation that the actin cytoskeleton was one of the principal systems-level targets of dasatinib, it did not identify any specific candidates that may be responsible for the unique phenotype we observe in podocytes. For example, kinase enrichment of the downregulated phosphoproteins showed that *SRC* was the highest associated upstream kinase, most likely due to its over-representation in the literature. However, looking at SRC activity across different KI treatments in podocytes showed that bosutinib treatment resulted in similar levels of inhibition, suggesting that one or more other upstream signaling pathways must have been exclusively impacted by dasatinib (but not by other KIs).

In order to identify kinases targeted by dasatinib (compared to the other tested KIs) for induction of the unique cytoskeletal phenotype, we used the previously published kinome-profiling database that quantified the catalytic activity of 300 human kinases under small molecule inhibition^18^. When the database was limited to include kinases that were inhibited >50% by one or several of these six tested KIs, dasatinib did not have a particularly different kinase inhibition signature (Supplementary Fig. 12). Given the experimental findings, we filtered the kinome-profiling dataset to retain targets that are associated with actin-related ontological terms as enriched in our proteomic analyses. We obtained 12 kinases (EPHA5, PDGFRB, PDGFRA, EPHA3, ABL2, ABL1, HCK, LIMK1, FES, PAK3, LYN, LRRK2) for which one or more of the six investigated KIs showed relevant inhibitory activity. Dasatinib showed the highest overall inhibitory affect (Fig. 5d). Furthermore, we noted that LIM kinase (LIMK1) was the only kinase that was inhibited by dasatinib alone and not by any of the other tested KIs, suggesting a potential role in the observed cytoskeletal phenotype.

LIM kinase is one of the key regulators for the formation and crosslinking of actin stress fibers through Rac/Cdc42 signaling^19^. Both its upstream activator PAK1/2/3^20^ and downstream effector cofilin^21^ have been shown to play key roles in maintaining podocyte FP architecture^22,23^. To test whether dasatinib uniquely inhibited LIM kinase along the Rac/Cdc42 pathway, we assayed the activity of PAK1, LIMK1 and cofilin in podocytes treated with the panel of six KIs for one hour. Indeed, western blot analysis showed that phosphorylation levels for both LIMK1 and cofilin-1 were significantly reduced only in dasatinib treated samples compared to podocytes treated with other KIs (Fig. 5e). In agreement with the proteomic enrichment analyses, we saw that PAK1 was also uniquely reduced in dasatinib treated podocytes, confirming that upstream regulatory pathways, including Rac/Cdc42 small GTPase signaling, were downregulated by dasatinib at a systems-level.

### Diminished cytoskeletal integrity leads to FP effacement

**Fig. 6:**
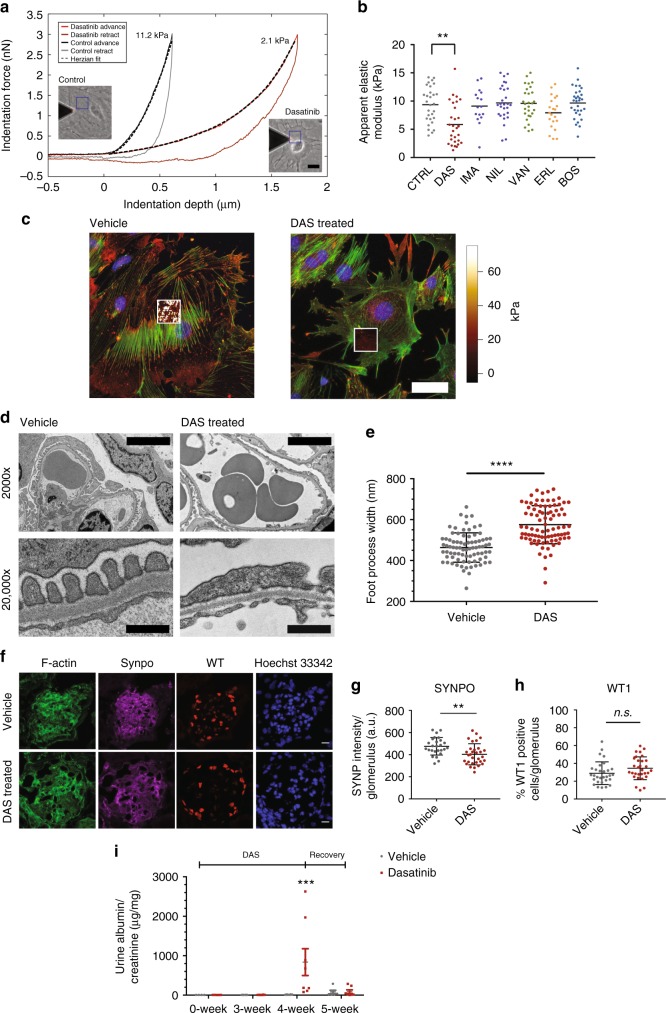
Dasatinib impacts podocyte biomechanics and induces foot process effacement in vivo. **a** Representative AFM indentation curves from elastography measurements of control and dasatinib treated podocytes that were used to quantify the single-cell biomechanical properties show good agreement between the Hertzian fits and the advance-phase of the raw indentation curves (insets: phase contrast images of representative cells with the superimposed blue line denoting the probed area; scale bar = 20 µm). **b** Significant reduction of apparent elastic modulus is observed in dasatinib-treated podocytes but not in other KI treatments (line: mean; **p < 0.01 dasatinib vs. all other groups, one-way ANOVA with post-hoc Tukey test). **c** Spatial AFM elastography maps highlight stiff linear projections that colocalize with the underlying F-actin stress fibers in control but not in dasatinib treated podocytes suggesting that existing fibers have lost their biomechanical integrity. **d** Representative TEM images of kidney sections for 129S1/SvImJ mice that have been treated daily with dasatinib for five weeks (*n* = 8, each group) show substantial FP effacement. Scale bars are 5000 and 500 nm for the 2000× and 20,000× images, respectively. **e** Quantification of FP width from TEM images (mean ± SD; ****p < 0.0001, unpaired *t*-test). **f** Immunofluorescence analysis shows that synaptopodin crosslinked actin fibers are reduced in dasatinib treated glomeruli, even though total number of podocytes is unchanged. Quantification of **g** synaptopodin (SYNPO) expression (mean ± SD; **p < 0.01, unpaired *t*-test) and **h** WT1-positive podocyte count per glomerulus as determined by WT1-positive cells show that F-actin crosslinking is significantly lower even though the number of podocytes do not change (mean ± SD; n.s. = not significant, unpaired *t*-test) in dasatinib treated animals. Scale bars = 10 µm. **i** 8-week old MRL/MpJ Lupus mice chronically treated with dasatinib show severe proteinuria within four weeks that recovers within a week upon suspension of treatment (mean ± SEM; ***p < 0.001, two-way ANOVA with Bonferroni multiple comparison)

Given LIM kinase and cofilin pathway’s direct role in maintaining the mature podocyte cytoskeleton^22^, we hypothesized that dasatinib would diminish the structural integrity of crosslinked stress fibers, which would lead to reduced biomechanical stiffness or cellular elasticity. We used our previously reported atomic force microscope (AFM) elastography technique^24^ to characterize the spatial distribution of cellular elasticity of podocytes under various KI treatments (Fig. 6a). As hypothesized, we found that only podocytes treated with dasatinib exhibited significant and robust reduction in their mean cellular elasticity (Fig. 6b and Supplementary Fig. 13); no other KI had a significant effect (*p* < 0.01 dasatinib vs. all other groups, one-way ANOVA with post-hoc Tukey). We observed that the reduction in depth-dependent pointwise elasticity was thoroughly evident through the axial dimension in further agreement with our HCA measurements for impaired stress fiber architecture (Supplementary Fig. 14). We also acquired AFM elastography maps with increased resolution (Supplementary Fig. 15), which when superimposed over immunostained high-resolution images of the F-actin for control podocytes, showed clear colocalization of actin stress fibers and the highly stiff heterogeneous elasticity measurements (Fig. 6c; left panel). This correlation was completely abolished under dasatinib treatment, which resulted in homogeneous, weak elasticity values (Fig. 6c; right panel). These observations also agreed with the recent reports showing that dasatinib significantly affected cellular biomechanics and adhesion of BRAF V600E mutant melanoma A375 cells^25^.

Our in vitro measurements of reduced cytoskeletal integrity suggested that chronic administration of dasatinib should induce foot process effacement in vivo. To test this, 8-week-old wild-type 129S1/SvImJ mice (*n* = 8, each group) were administered either with clinically relevant doses of dasatinib or vehicle control once a day through oral gavage for five weeks. Ultrastructurally, dasatinib treated mice showed FP effacement (Fig. 6d) and significant increase in lateral FP width (Fig. 6e). To assess the integrity of the podocyte actin cytoskeleton in vivo, we used immufluorescence measurements looking at synaptopodin-crosslinked actin stress fibers (Fig. 6f). In agreement with the in vitro observations of a substantial cytoskeletal effect with little change in cellular viability, we saw significant reduction in synaptopodin expression in dasatinib treated mice (Fig. 6g) with no change in podocyte number as quantified by nuclei positive for the podocyte-specific marker WT1 (Fig. 6h). We also noted that LIMK1 phosphorylation was reduced, confirming that Rac/Cdc42 pathway was impacted in vivo by dasatinib leading to glomerular dysfunction (Supplementary Fig. 16). Formalin-fixed paraffin embedded tissue sections were stained with hematoxylin-eosin (H&E), Periodic Acid Schiff (PAS) and trichrome and evaluated by a blinded expert renal pathologist. Histology examination showed no significant tubular morphology changes or discernable differences between animals treated with vehicle or dasatinib (Supplementary Fig. 17a-c). These observations further mirrored the FAERS risks scores, which suggested relatively low tubular toxicity for dasatinib. As an in vitro quality control step, we also performed MTT-based viability assays on cultured mouse tubular epithelial cells, which showed that dasatinib had an equivalent or comparable impact on viability of tubular epithelia with respect to the other KIs tested and that this was similar to that observed on podocytes (Supplementary Fig. 17D). While there were no significant tubular effects, some of the mice showed mild and irregular proteinuria at five weeks, even though the effect was not significant (Supplementary Fig. 18). The mild injury phenotype was consistent with clinical observations of general tolerability, which further suggested that clinical cases might be due to repeated sub-threshold injury in susceptible patients. To further test this hypothesis, we chronically administered dasatinib in lupus nephritis MRL mice, where podocytes have been shown to display compromised actin cytoskeleton^26,27^. Accordingly, our hypothesis would predict that these animals would be more susceptible to cytoskeletal injury, and thus they would exhibit markedly increased glomerular dysfunction when challenged by dasatinib. In fact, when these mice were treated daily with dasatinib, they developed severe proteinuria within four weeks, which was completely reversed after a week of suspending drug administration (Fig. 6i).

## Discussion

Understanding the mechanisms of drug toxicity is a central research goal in clinical oncology. The drug effects on cell survival and proliferation pathways are often considered as key parameters when evaluating safety and efficacy. In this study, we use an integrative approach to show that the impact of a drug on cell biophysics could also be relevant to understanding its ADR. Dasatinib, a second-generation BCR-ABL1 inhibitor, destabilizes the actin cytoskeleton of kidney podocytes through its systems-level action that includes impairment of FA architecture and PAK-LIMK signaling. Cytoskeletal damage has been suggested as a mechanism of drug toxicity as early as 1990s^28^. While cytoskeletal effects of toxins may play a limited role in the survival of most cell types, actin-associated proteins serve a critical function in maintenance of the structural integrity of kidney podocytes^29^ that have been shown to have exceptionally fragile cytoskeletal dynamics^30^; therefore, it is important to examine the effects of new therapeutics on the podocyte cytoskeleton. Podocytes perform their primary function under constant mechanical stress; in fact, loss of podocytes due to biomechanical injury has been suggested as a key metric in disease progression that may be even more appropriate than proteinuria^31^. Our findings show that the direct effect of a toxin on podocyte cytoskeleton could increase the likelihood of nephrotoxic ADR even in the absence of other biomechanical risk factors, such as hypertension.

We used the FAERS database to quantify relative risk scores for glomerulopathies and hypertension associated with kinase inhibitors. The FAERS database has limitations, the most important limitation being that it does not allow absolute quantification of ADR risk but only relative to other ADR reported for various drugs^32^. In order to make a more controlled within-class comparisons, we have chosen to only compare relative ADR risks among other small molecule KI drugs in the FAERS database. The obtained RORs served as a starting point to perform the mechanistic studies described in this report. The absence of an increase in risk for hypertension in dasatinib patients was one of the critical observations that suggested a direct link between podocytes and the nephrotoxic ADR. The disconnect between increased risk for proteinuria in the absence of increased biomechanical stress supports the hypothesis that podocytes are structurally weaker and more prone to biophysical damage when treated with dasatinib, even under normal physiological conditions. Here, we used atomic force microscopy to show that dasatinib uniquely affected the structural integrity of the podocyte cytoskeleton leading to decreased cellular elasticity that impacts its physiological function as a structural member of the filtration barrier. We also recapitulated this pathophysiological effect in vivo using chronic administration of dasatinib in mice, which resulted in dysregulation of the actin cytoskeleton and FP morphology.

Even though proteinuria was observed in a few patients on the highest dose of dasatinib during its phase I trial^33^, at the time of this study, dasatinib was not considered a nephrotoxic therapeutic. Following its initial FDA approval, case reports of nephrotic range proteinuria^9^ and acute renal failure^34^ had been presented. The authors of these reports, respectively, postulated that possible off-target effects of dasatinib against VEGF and PDGF receptor might be responsible. While dasatinib has mild inhibitory activity against the PDGF receptor^35^, we noted that other KIs with stronger inhibition of either receptor tyrosine kinases did not affect the adhesion, morphology, or the cytoskeleton of cultured podocytes. The kinome profiling comparisons further corroborated these findings, suggesting that none of the growth factor receptor tyrosine kinases were uniquely affected by dasatinib (e.g., PDGF receptor activity was inhibited equally by imatinib and nilotinib; ephrin signaling was equally impacted by nilotinib and bosutinib; VEGF receptor activity was reduced mostly by vandetinib followed by bosutinib). Instead, we saw that kinases associated with the Rac/Cdc42 pathway and actin regulation were mostly influenced by dasatinib. In particular, we showed that PAK-LIMK signaling axis was significantly, and exclusively, impacted by dasatinib leading to reduced cofillin phosphorylation and actin stress fiber formation.

A healthy actin cytoskeleton is crucial for the maintenance of morphological and biophysical properties of the eukaryotic cell^36^. In addition to its role in structural integrity, actin plays important roles in mechanosensation^37^, endocytosis^38^, and transcriptional regulation^39^. Here, we show that dasatinib impacts a vast interconnected signaling network that focuses on the control of focal adhesion architecture and the actin cytoskeleton, which are critical for podocyte physiology^40^. It is important to note that the effect is not singular but at a systems-level. For example, in addition to the Rac/Cdc42 pathway, dasatinib also had a severe effect on Hippo signaling, as measured through nuclear YAP translocation (Supplementary Fig. 19). We recently showed that regulators of YAP spatial activity, such as KIBRA, play a critical role in organization of the actin cytoskeleton in podocytes^41^. Recently, biomechanical integrity of podocytes had been proposed as a coherent signature that underlies multiple glomerular disease models^42^, supporting our hypothesis that drugs that challenge the actin cytoskeleton architecture in podocytes may induce glomerular dysfunction and nephrotoxicity. We also note that while our in vitro assays were extensive, covering a varying range of time and concentration values for dasatinib and the other KIs, the dynamics of the podocyte kinome that controls the actin cytoskeleton is clearly complex, and it needs to be studied in further detail.

We acknowledge that the in vivo functional effect of dasatinib in young and healthy wild-type 129S1/SvImJ mice was subtle yet robust; it was recognizable as consistent major ultrastructural alterations with little change in proteinuria. This observation also agreed with prior literature that has showed that in subclinical phenotypes, such as minimal change disease, ultrastructural damage to podocyte FP precedes major glomerular dysfunction^43–45^. This is also consistent with the odds ratios we have observed in our clinical meta-analyses and the low number of nephrotic range proteinuria case studies in the literature. The nephrotoxic effect of dasatinib at its clinically used doses is clearly tolerable and non-toxic to the renal tubules; however, it is imperative to note that (1) susceptible individuals (or those who are at risk due to environmental factors) may be more likely to suffer from a cytoskeletal insult, and that (2) even subclinical impairment of the cytoskeleton increases podocytes’ vulnerability to further injury. Our results in the lupus mice further support this second hit hypothesis suggesting that dasatinib induces a cytoskeletal challenge that critically impacts podocyte function, but not immediate survival since the massive proteinuria induced by dasatinib was quickly reversed once treatment was discontinued. This is in agreement with case studies that have observed immediate resolution of proteinuria in patients, where dasatinib was replaced with another BCR-ABL1 inhibitor^9^.

It is well known that as podocytes are postmitotic, acute injuries greatly increase the risk of CKD and other co-morbidities^6^ even if they were completely resolved^46^. Subclinical nephrotoxicity of dasatinib is particularly important because CML small-molecule regimens are increasingly becoming chronic long-term treatments that last for decades. In this regard, these observations carry additional significance since small subthreshold injury sustained during dasatinib treatment may reflect as increased risk of developing CKD later in life. We therefore conclude that patients on dasatinib should be routinely checked for proteinuria and immediately referred for nephrology evaluation if proteinuria is observed.

## Methods

### Clinical meta-analyses

Adverse event frequencies from the FDA Adverse Event Reporting System (FAERS) were obtained from the AERSmine resource that contains a curated version of the FAERS database^47^. ADR in the FAERS database are organized according to MedDRA terms^48^, which belong to a hierarchical ontology to classify ADR from high-level organs associated with the pathology to reported low-level specific conditions. Our ADR risk analysis was limited to FDA-approved protein kinase inhibitors. We downloaded the frequencies of occurrence of renal ADR for all protein KIs available in FAERS, together with all other frequencies of ADR reported for these KIs between the years 2004–2018. A time-stamped record of this download to reproduce this analysis was retained and it is included in Supplementary Data 1 along with the scripts to reproduce this analysis. Reporting odds ratios (RORs) were then computed for each KI using the frequency *f*_dt_ of the ADR of interest (i.e., glomerulonephritis, nephrotic syndrome, hypertension) in patients using the KI of interest, the frequency *f*_dn_ of any other ADR occurring in patients using the KI of interest, the frequencies *f*_nt_ of occurrence of the ADR of interest (i.e., glomerulonephritis, nephrotic syndrome, hypertension) for any other protein kinase inhibitor, and the frequency *f*_nn_ for all other ADR and all other KIs. The ROR was calculated using

1 $${\mathrm{ROR}} = \frac{{f_{{\mathrm{dt}}}/f_{{\mathrm{dn}}}}}{{f_{{\mathrm{nt}}}/f_{{\mathrm{nn}}}}},$$

whereas the standard error (SE) of the log(ROR) was calculated as

2 $${\mathrm{SE}}_{{\mathrm{log(ROR)}}} = \sqrt {\frac{1}{{f_{{\mathrm{dt}}}}} + \frac{1}{{f_{{\mathrm{dn}}}}} + \frac{1}{{f_{{\mathrm{nt}}}}} + \frac{1}{{f_{{\mathrm{nn}}}}}},$$

with the log-transformed 95% confidence interval (CI) being calculated as

3 $${\mathrm{CI}} = \log \left( {{\mathrm{ROR}}} \right) \pm 1.96 \times {\mathrm{SE}}_{\log \left( {{\mathrm{ROR}}} \right)}.$$

The resulting RORs and confidence intervals were visualized for the KIs studied experimentally (color-coded universally across the paper), with additional commonly used KIs that have been FDA approved.

### MTT viability assay

Conditionally immortalized mouse podocytes (gift from Peter Mundel^49^) or tubular epithelial cells (gift from Luca Gusella^50^) were seeded on clear tissue culture-treated 96-well plates. Podocytes were differentiated for 10 days at 37 °C, while tubular cells were cultured for 48 h prior to testing. Cells were then treated with one of the selected KIs at a concentration ranging from 0.5 nM to 50,000 nM for 24 h at a final volume of 100 µl. 5 mg/ml 3-(4, 5-dimethylthiazolyl-2)-2, 5-diphenyltetrazolium bromide was dissolved in PBS and sterile filtered. During the last 4 h of drug treatment, 10 µl of MTT solution were added to each well at 37 °C. After completion of incubation, MTT-supplemented media were replaced with 100 µl of DMSO per well, plates were gently shaken on an orbital shaker for one minute, and absorbance was measured at 500 nm on a Molecular Devices DTX800 plate reader at 25 °C.

### High-content image analyses (HCA)

Morphometric analyses were carried out as described previously^41,51^ with modifications. Conditionally immortalized podocytes were seeded on black, clear-bottomed tissue culture-treated 96-well assay plates (Corning Incorporated) or black glass-bottom 96-well assay plates (Nunc) at a density of 800–1000 cells/well. After culturing at 37 °C for 10 days, podocytes were treated with the appropriate drug or DMSO vehicle control (diluted at 1:1667 or more). Podocytes were fixed with 4% paraformaldehyde in PBS for 15 min at room temperature, washed three times with PBS, permeabilized with 0.1 % Triton X-100 for 10 min and blocked for 1 h with 5% normal goat serum supplemented with 0.05% Triton X-100. Cells were then incubated with primary antibodies in blocking buffer at 4 °C overnight, washed with PBS and incubated with secondary antibodies (AlexaFluor 488, AlexaFluor 568 and/or AlexaFluor 647, Thermo Fisher) at 1:500 dilution in blocking buffer for one hour at room temperature and subsequently counterstained for F-actin and nucleus with ActinGreen 488 (Thermo Fisher) and Hoechst 33342, respectively, according to manufacturer’s specifications. Image acquisition was carried out on an InCell Analyzer 2200 (GE Healthcare) using 20× and 40× air objectives for cell and focal adhesion imaging, respectively. Image analysis and quantification were performed using ImageJ and Broad Institute’s CellProfiler suite^52^. Briefly, nuclear objects were identified in the Hoechst 33342 channel, and the corresponding cell objects were identified using the innate propagation algorithm of CellProfiler coupled with the contrast-enhanced phalloidin channel to define cell boundaries. Multi-parametric analysis was performed where at least 60 features were obtained from the four channel images including intensity, texture, shape and size metrics. Measurements were exported directly to csv files and subsequently analyzed using MATLAB to generate histograms and distribution plots. In addition to the MTT assay we also used the total nuclear counts per well as a second measure of cell viability. Detailed information on all primary antibodies is available in Supplementary Table 5.

### Immunofluorescence staining

Frozen kidney sections were fixed in 4% paraformaldehyde, permeabilized in 0.1% Triton X-100 for 10 min and blocked for one hour with 5% normal goat serum supplemented with 0.05% Triton X-100. Sections were incubated with the primary antibodies in blocking solution, overnight at 4 °C (parameters for the antibodies are listed in Supplementary Table 5). Sections were subsequently washed with PBS and incubated with secondary antibodies at 1:250 dilution in blocking buffer (AlexaFluor 568 and AlexaFluor 647, Thermo Fisher Scientific) at one hour at room temperature. Kidney sections were then stained for F-actin and nucleus with ActinGreen 488 (Thermo Fisher Scientific) and Hoechst 33342, respectively. Cells were then imaged on a Zeiss LSM 880 (Jena, Germany) equipped with 20× and 40× objectives. Across all samples, same acquisition settings were applied (laser power, gain settings, magnification, zoom, pixel size, slice thickness and number of slices). Image quantification was performed on maximum intensity projections of Z-stacks, on ImageJ. To obtain synaptopodin intensity/glomeruli, regions of interest (ROI) on the corresponding channel was chosen by using the thresholded and binarized regions of the actin channel.

### Immunoblot analysis

Conditionally immortalized mouse podocytes, cultured on 10 cm culture dishes for 10 days at 37 °C and treated with vehicle control or the selected KIs for 24 h, were lysed with 4 °C RIPA buffer (Boston Bioproducts) supplemented with 2× HALT protease and phosphatase inhibitors (Thermo Fisher Scientific) on ice. After measurement of protein concentration, 6× loading buffer with SDS (Boston Bioproducts) was added, and samples were boiled at 95 °C for 5 min. After equilibrating concentrations, 35 μg of protein per sample was then resolved in SDS-PAGE with 4–20% gradient gels (Bio-rad) and wet transferred into nitrocellulose membranes (Bio-Rad) with a constant current of 30 mA at 4 °C overnight. Membranes were then washed, blocked with casein blocking buffer for one hour, incubated overnight with primary antibodies, washed with TBS buffer, incubated with IR-fluorescence secondary antibodies (Li-Cor) for one hour, washed with TBS buffer, and visualized on a Li-Cor Odyssey CLx imaging system. Intensity of appropriate bands were quantified and normalized to those of loading control bands using the Li-Cor Image Studio software suite according to manufacturer’s instructions. Normalized intensities from at least three independently-run biological replicates were statistically compared using unpaired *t*-tests. Detailed information on all primary antibodies is available in Supplementary Table 5. Full size, uncropped images of all the western blots used in this study are shown in Supplementary Fig. 20.

### Phospho-tyrosine enriched proteomics

Conditionally immortalized mouse podocytes were plated on 15 cm culture dishes at a concentration of 2000 cells per cm^2^. After 10 days of differentiation at 37 °C, cells treated for 1 h with 2 μM dasatinib or DMSO vehicle control (*n* = 3, each) were lysed in immunoprecipitation (IP) lysis buffer containing 150 mM NaCl, 1 mM EDTA, 10% glycerol, 0.1% Tween 20, and 50 mM HEPES pH 7.4. To enrich for tyrosine-phosphorylated proteins, 0.5 mg total protein from each concentration-normalized lysate were incubated with 8 μg anti-mouse ferromagnetic beads pre-coated with mouse anti-phospho-tyrosine primary antibodies at 4 °C overnight on a rotating carousel. The beads were washed five times with 1 M NaCl with 0.1% Tween 20 at 4 °C and then eluted using 40 μl of 2× loading buffer (Boston Bioproducts) and boiled at 95 °C for 5 min. In order to remove contamination that could affect the liquid chromatography-tandem mass spectrometry (LC-MS/MS) analysis, the eluted IP proteins were separated on 10% SDS-PAGE for in-gel trypsin digestion.

The gel lane of each sample was excised and washed with 30% ACN in 50 mM ammonium bicarbonate prior to DTT reduction and iodoacetamide alkylation. After in-gel trypsin digestion at 37 °C overnight, the resulting peptides were subjected to LC-MS/MS analysis on ultimate 3000 LC system coupled with a Q Exactive tandem mass spectrometry instrument (Thermo Fisher). In brief, the peptides were separated by a C18 reversed phase column (75 μm × 50 cm, 2 μm, 100 Å, C_18_, Thermo Fisher) using a 205-min binary gradient (solvent A (2% acetonitrile (ACN), 0.1% formic acid (FA)), solvent B (85% ACN, 0.1% FA): 10 min gradient from 1% to 5% B followed by 165 min gradient from 5% to 30% B, 15 min gradient from 30% to 50% B, and 15 min gradient from 50% to 95% B). The eluted peptides were directly introduced into a nanospray Flex ion source on Q Exactive mass spectrometry system with a spray voltage of 2.15 kV and a capillary temperature of 275 °C. Initial spectra were acquired in a positive ion mode with the MS mass range between *m*/*z* 400–1700. The resolution was set to 140,000 FWHM for MS and 17,500 for MS/MS. The automatic gain control (AGC) target was set to 3 × 10^6^ for full scan and 5 × 10^5^ for MS/MS scan in Orbitrap mass analyzer. The precursor isolation width was 2 *m*/*z*.

The MS/MS spectra were searched against the Swiss-Prot mouse database (16,571 sequences) using Mascot (V2.3) search engine on Proteome Discoverer (V1.4) platform. The mass error tolerance was 10 ppm for MS and 0.1 Da for MS/MS. Methionine oxidation, phosphorylation of tyrosine and cysteine carbamidomethyl (IAM) modification were set as variable modifications. The protein homologs were further grouped in Scaffold software (Proteome Software, Inc) to remove redundancies. Acceptable protein false discovery rate cut off was less than 1%. Dasatinib treated samples were compared to vehicle-treated control samples using the spectra counting method^16,53^, and differentially expressed proteins were identified using the Benjamini-Hochberg procedure^54^. Top 76 downregulated proteins (with *p*-values < 0.05) were unsupervised clustered using Euclidean distance, and then connected within the human protein-protein interactome using X2K with one intermediate node that was vetted by a minimum of two literature sources excluding the yeast two-hybrid studies^55^. Gene set, pathway and ontological enrichment was carried out using the EnrichR suite^17^. The experiment was repeated twice with three biological replicates each time. Summary results of the repeat phospho-proteomic assay were similar as shown in Supplementary Fig. 21.

### Kinome-profiling analyses

We used the publicly available kinome profiling data from Anastassiadis et al.^18^ that contained inhibition profiles of 300 kinases treated with 178 clinically approved or experimental small molecules. The dataset was first filtered by removing all protein kinases that exhibited more than 50% residual activity across all KIs, i.e., to remove any protein kinases with no relevance. We then further filtered the kinome database to only retain kinases with an established relationship to actin-related processes. For this, we used the Gene Ontology Biological Processes database, where we identified and aggregated all actin-related terms and associated genes. We calculated mean residual activity for each drug across the remaining kinases and visualized both individual residual kinase activities under each drug as well as the mean residual activity.

### Atomic force microscope elastography

Elastography measurements and analyses were carried out as previously described^56^ using an Asylum MFP-3D-BIO atomic force microscope coupled with an Olympus IX-80 inverted spinning disk confocal microscope. Briefly, conditionally immortalized mouse podocytes were plated on collagen type-I coated 50 mm short-profile plastic dishes (BD) and cultured for 10 days under differentiation conditions at 37 °C. They were then treated with 2 μM of one of the six KIs (dasatinib, imatinib, nilotinib, vandetanib, erlotinib, bosutinib) or vehicle control for 30–45 min at 37 °C. A 0.1 N/m gold-coated silicon nitride blunt pyramidal AFM tip (Cat #: TR400PB, Asylum) was placed on the liquid probe holder and calibrated using the thermal noise method. Cells, treated with DMSO (vehicle) or KIs, were placed on the AFM stage at 33 °C (lower temperature was used during experimentation to reduce evaporation and instrument drift; even though we did not see any statistical differences between differentiated podocyte indentation experiments performed at 37 °C with those performed at 33 °C, we note that this lower temperature may have a small effect on cellular biomechanics). After 15 min of thermal equilibrium, 20 μm perinuclear region of podocytes were probed with a resolution of 4 μm using a 6 × 6 homogeneous array of 4 μm deep indentations (at 8 μm/s) with a uniform trigger threshold of 30 nm. No more than ten cells were probed for each dish, limiting the assay time on the instrument to 45 min. Contact point and depth-dependent pointwise apparent elastic modulus were computed from each indentation curve as previously described^57^; here, we report both median Hertzian fitted elastic modulus as the homogenized cellular elasticity measurement for the given cell and the depth-dependent pointwise apparent elastic modulus. Since increased aberrant virtual stiffening is a common concern for AFM elasticity measurements in thin specimens^58,59^, we checked both goodness of Hertzian fit (Fig. 6a) and the depth-dependence of our pointwise elastic modulus, which was shown to be a sensitive marker for substrate effects^60^. These measurements showed that our indentations were not affected by the substrate effect (Supplementary Fig. 14). For spatial elastography assays with increased resolution, the above measurement protocol was modified for a spatial resolution of 1 μm by performing an indentation array of 32 × 32 over an area of 30 × 30 μm. After the AFM assay, cells were fixed with 4% paraformaldehyde and immunostained for paxillin and actin as described above. The experiment was repeated four times independently whereby all drug conditions were tested at each given time and the testing order of drugs were randomly chosen.

### In vivo mouse experiments

All animal received humane and ethical care in compliance with the “Principles of Laboratory Animal Care” as outlined by the NIH Publication No. 85-23 (revised 1985). In addition, all animal studies were approved by the Institute for Animal Care and Use Committee at Icahn School of Medicine at Mount Sinai. 8-week old 129S1/SvImJ mice (*n* = 8) from Jackson Labs were gavaged daily with either 20 mg/kg body weight dasatinib in citrate buffer or vehicle control for five weeks. After five weeks of daily gavages, animals were anesthetized and perfused intraventricularly with 50 ml of sterile filtered 3% paraformaldehyde in PBS. The kidneys were removed and processed for histology and electron microscopy as previously described^61^. Histopathological analysis was performed by a blinded expert renal pathologist using Periodic acid–Schiff (PAS), Hematoxylin-eosin, and Masson’s trichrome stains to evaluate general morphology of the basement membrane, tubular morphology, and extent of fibrosis, respectively. To assess the reversibility of dasatinib induced proteinuria and test our second-hit hypothesis, 8-week old MRL/MpJ-*Fas*^*lpr*^ lupus mice (*n* = 8) from Jackson Labs were gavaged daily either with 20 mg/kg body weight dasatinib in citrate buffer or vehicle control for four weeks after which treatment was discontinued and animals were allowed to recover for an additional week. Urine was collected every other day. Urine albumin was measured according to the manufacturer’s protocol using the mouse albumin ELISA quantitation set from Bethyl Laboratories (Montgomery, TX), whereas urine creatinine was measured according to the manufacturer’s protocol with the creatinine colorimetric assay kit (Cayman Chemicals, Ann Arbor, MI).

### Quantitative transmission electron microscopy

In vivo FP morphometrics were analyzed as previously described^61^. Briefly, after perfusion of the fixative, kidneys were dissected; 5 mm cubic cortical segments were cut, and further fixed with 2.0% glutaraldehyde in 0.2 M sodium cacodylate for 48 h. The samples were then washed with phosphate buffer and osmicated with 1% osmium tetroxide for 1 h. After quick rinse in phosphate buffer and three 10-minute incremental ethanol dehydration steps, samples were washed with propylene oxide three times and stained in the dark with uranyl acetate-lead citrate for 1-h. They were then embedded in epon resin, and 80 nm thin sections were prepared. Thin sections were imaged on a fully digital Hitachi H7600 transmission electron microscope at 75 kV. Glomerular segments were imaged at a low 2000× resolution for quantification of FP width and at a high 20,000× resolution for visualization of the slit diaphragm architecture. For quantification, five or more glomeruli per animal were imaged randomly over at least two separate thin sections. The number of cell-cell junctions per linear capillary distance was measured in a blinded fashion using Image J.

### Statistical methods

For high content analysis, nonparametric Kruskal–Wallis one-way analysis of variance (ANOVA) followed by post-hoc Tukey multiple comparison tests were used, and a *p*-value of 0.001 was considered statistically significant. For all other experiments, significance was achieved at a *p*-value of 0.05. Statistical comparisons for western blots, MTT viability assays, electron microscopic morphometry, urine albumin-creatinine ratio in wild-type animals, and immunofluorescence staining for the in vivo experiments were performed with unpaired t-tests. For evaluation of cellular stiffness with atomic force microscope elastography, one-way ANOVA with post-hoc Tukey test was used. For the timecourse evaluation of urine albumin-creatinine ratio in Lupus mice, a two-way ANOVA with Bonferroni multiple comparison post-hoc test was used.

All imaging experiments and high-content assays were performed with at least three well replicates each time. Every experiment (including the animal experiments) was repeated at least three times using brand new biological material. Due to its high cost, phosphoproteomics experiment was repeated twice with three independent biological replicates each time.

### Reporting summary

Further information on research design is available in the Nature Research Reporting Summary linked to this article.

## Supplementary information

Supplementary Info

Description of Additional Supplementary Files

Supplementary Data 1

Reporting Summary

## Data Availability

All data supporting the findings of this study are available from the corresponding author upon reasonable request. Raw mass spectrometry proteomics data have been deposited to the ProteomeXchange Consortium via the PRIDE partner repository with the dataset identifier PXD011761 and [https://www.ebi.ac.uk/pride/archive/projects/PXD011761].

## Code availability

All the annotated data mining and image analysis protocols and codes are available within the supplementary information files and Azeloglu Lab GitHub page [https://github.com/AzelogluLab], respectively.

## References

[1] Wheler JJ (2012). Risk of serious toxicity in 1181 patients treated in phase I clinical trials of predominantly targeted anticancer drugs: the M. D. Anderson Cancer Center experience. Ann. Oncol..

[2] Wong G (2009). Association of CKD and cancer risk in older people. J. Am. Soc. Nephrol..

[3] Launay-Vacher V (2015). Renal effects of molecular targeted therapies in oncology: a review by the Cancer and the Kidney International Network (C-KIN). Ann. Oncol..

[4] Abbas A, Mirza MM, Ganti AK, Tendulkar K (2015). Renal toxicities of targeted therapies. Target Oncol..

[5] Downing NS (2017). Postmarket safety events among novel therapeutics approved by the US Food and Drug Administration between 2001 and 2010. JAMA.

[6] Coca SG, Singanamala S, Parikh CR (2012). Chronic kidney disease after acute kidney injury: a systematic review and meta-analysis. Kidney Int..

[7] Zhao S (2013). Systems pharmacology of adverse event mitigation by drug combinations. Sci. Transl. Med..

[8] Law V (2014). DrugBank 4.0: shedding new light on drug metabolism. Nucleic Acids Res..

[9] Wallace E, Lyndon W, Chumley P, Jaimes EA, Fatima H (2013). Dasatinib-induced nephrotic-range proteinuria. Am. J. Kidney Dis..

[10] Eskens FA, Verweij J (2006). The clinical toxicity profile of vascular endothelial growth factor (VEGF) and vascular endothelial growth factor receptor (VEGFR) targeting angiogenesis inhibitors; a review. Eur. J. Cancer.

[11] Shankland SJ, Pippin JW, Reiser J, Mundel P (2007). Podocytes in culture: past, present, and future. Kidney Int..

[12] Chen J, Chen JK, Harris RC (2015). EGF receptor deletion in podocytes attenuates diabetic nephropathy. J. Am. Soc. Nephrol..

[13] Advani A (2011). Inhibition of the epidermal growth factor receptor preserves podocytes and attenuates albuminuria in experimental diabetic nephropathy. Nephrology.

[14] McCaig AM, Cosimo E, Leach MT, Michie AM (2012). Dasatinib inhibits CXCR4 signaling in chronic lymphocytic leukaemia cells and impairs migration towards CXCL12. PLoS One.

[15] Ron A (2017). Cell shape information is transduced through tension-independent mechanisms. Nat. Commun..

[16] Arike L, Peil L (2014). Spectral counting label-free proteomics. Methods Mol. Biol..

[17] Chen EY (2013). Enrichr: interactive and collaborative HTML5 gene list enrichment analysis tool. BMC Bioinform..

[18] Anastassiadis T, Deacon SW, Devarajan K, Ma H, Peterson JR (2011). Comprehensive assay of kinase catalytic activity reveals features of kinase inhibitor selectivity. Nat. Biotechnol..

[19] Yang N (1998). Cofilin phosphorylation by LIM-kinase 1 and its role in Rac-mediated actin reorganization. Nature.

[20] Edwards DC, Sanders LC, Bokoch GM, Gill GN (1999). Activation of LIM-kinase by Pak1 couples Rac/Cdc42 GTPase signalling to actin cytoskeletal dynamics. Nat. Cell Biol..

[21] Arber S (1998). Regulation of actin dynamics through phosphorylation of cofilin by LIM-kinase. Nature.

[22] Garg P (2010). Actin-depolymerizing factor cofilin-1 is necessary in maintaining mature podocyte architecture. J. Biol. Chem..

[23] Zhu J (2010). p21-activated kinases regulate actin remodeling in glomerular podocytes. Am. J. Physiol. Ren. Physiol..

[24] Azeloglu EU, Bhattacharya J, Costa KD (2008). Atomic force microscope elastography reveals phenotypic differences in alveolar cell stiffness. J. Appl Physiol..

[25] Logue JS, Cartagena-Rivera AX, Chadwick RS (2018). c-Src activity is differentially required by cancer cell motility modes. Oncogene.

[26] Liao R (2015). Tacrolimus protects podocytes from injury in lupus nephritis partly by stabilizing the cytoskeleton and inhibiting podocyte apoptosis. PLoS One.

[27] Trivedi S, Zeier M, Reiser J (2009). Role of podocytes in lupus nephritis. Nephrol. Dial. Transpl..

[28] Bellomo G (1990). The cytoskeleton as a target in quinone toxicity. Free Radic. Res. Commun..

[29] Faul C, Asanuma K, Yanagida-Asanuma E, Kim K, Mundel P (2007). Actin up: regulation of podocyte structure and function by components of the actin cytoskeleton. Trends Cell Biol..

[30] Falkenberg CV (2017). Fragility of foot process morphology in kidney podocytes arises from chaotic spatial propagation of cytoskeletal instability. PLoS Comput Biol..

[31] Yu D (2005). Urinary podocyte loss is a more specific marker of ongoing glomerular damage than proteinuria. J. Am. Soc. Nephrol..

[32] Sakaeda T, Tamon A, Kadoyama K, Okuno Y (2013). Data mining of the public version of the FDA adverse event reporting system. Int J. Med Sci..

[33] Demetri GD (2009). Phase I dose-escalation and pharmacokinetic study of dasatinib in patients with advanced solid tumors. Clin. Cancer Res..

[34] Holstein SA, Stokes JB, Hohl RJ (2009). Renal failure and recovery associated with second-generation Bcr-Abl kinase inhibitors in imatinib-resistant chronic myelogenous leukemia. Leuk. Res..

[35] Lombardo LJ (2004). Discovery of N-(2-chloro-6-methyl- phenyl)-2-(6-(4-(2-hydroxyethyl)- piperazin-1-yl)-2-methylpyrimidin-4- ylamino)thiazole-5-carboxamide (BMS-354825), a dual Src/Abl kinase inhibitor with potent antitumor activity in preclinical assays. J. Med. Chem..

[36] Fletcher DA, Mullins RD (2010). Cell mechanics and the cytoskeleton. Nature.

[37] Luo T, Mohan K, Iglesias PA, Robinson DN (2013). Molecular mechanisms of cellular mechanosensing. Nat. Mater..

[38] Soda K, Ishibe S (2013). The function of endocytosis in podocytes. Curr. Opin. Nephrol. Hypertens..

[39] Miralles F, Visa N (2006). Actin in transcription and transcription regulation. Curr. Opin. Cell Biol..

[40] Perico L, Conti S, Benigni A, Remuzzi G (2016). Podocyte-actin dynamics in health and disease. Nat. Rev. Nephrol..

[41] Meliambro Kristin, Wong Jenny S., Ray Justina, Calizo Rhodora C., Towne Sara, Cole Beatriz, El Salem Fadi, Gordon Ronald E., Kaufman Lewis, He John C., Azeloglu Evren U., Campbell Kirk N. (2017). The Hippo pathway regulator KIBRA promotes podocyte injury by inhibiting YAP signaling and disrupting actin cytoskeletal dynamics. Journal of Biological Chemistry.

[42] Embry Addie E., Liu Zhenan, Henderson Joel M., Byfield F. Jefferson, Liu Liping, Yoon Joonho, Wu Zhenzhen, Cruz Katrina, Moradi Sara, Gillombardo C. Barton, Hussain Rihanna Z., Doelger Richard, Stuve Olaf, Chang Audrey N., Janmey Paul A., Bruggeman Leslie A., Miller R. Tyler (2018). Similar Biophysical Abnormalities in Glomeruli and Podocytes from Two Distinct Models. Journal of the American Society of Nephrology.

[43] Shankland SJ (2006). The podocyte’s response to injury: role in proteinuria and glomerulosclerosis. Kidney Int..

[44] van den Berg JG, van den Bergh Weerman MA, Assmann KJ, Weening JJ, Florquin S (2004). Podocyte foot process effacement is not correlated with the level of proteinuria in human glomerulopathies. Kidney Int..

[45] Tondel C (2015). Foot process effacement is an early marker of nephropathy in young classic Fabry patients without albuminuria. Nephron.

[46] Jones J (2012). Association of complete recovery from acute kidney injury with incident CKD stage 3 and all-cause mortality. Am. J. Kidney Dis..

[47] Sarangdhar M (2016). Data mining differential clinical outcomes associated with drug regimens using adverse event reporting data. Nat. Biotechnol..

[48] Brown EG, Wood L, Wood S (1999). The medical dictionary for regulatory activities (MedDRA). Drug Saf..

[49] Mundel P, Reiser J, Kriz W (1997). Induction of differentiation in cultured rat and human podocytes. J. Am. Soc. Nephrol..

[50] Lee K, Boctor S, Barisoni LM, Gusella GL (2015). Inactivation of integrin-beta1 prevents the development of polycystic kidney disease after the loss of polycystin-1. J. Am. Soc. Nephrol..

[51] Xie J (2015). Novel mutations in the inverted formin 2 gene of Chinese families contribute to focal segmental glomerulosclerosis. Kidney Int..

[52] Carpenter AE (2006). CellProfiler: image analysis software for identifying and quantifying cell phenotypes. Genome Biol..

[53] Zhou W, Liotta LA, Petricoin EF (2012). The spectra count label-free quantitation in cancer proteomics. Cancer Genom. Proteom..

[54] Benjamini Y, Hochberg Y (1995). Controlling the false discovery rate—a practical and powerful approach to multiple testing. J. R. Stat. Soc. B.

[55] Chen EY (2012). Expression2Kinases: mRNA profiling linked to multiple upstream regulatory layers. Bioinformatics.

[56] Azeloglu EU, Costa KD (2011). Atomic force microscopy in mechanobiology: measuring microelastic heterogeneity of living cells. Methods Mol. Biol..

[57] Azeloglu EU, Costa KD (2010). Cross-bridge cycling gives rise to spatiotemporal heterogeneity of dynamic subcellular mechanics in cardiac myocytes probed with atomic force microscopy. Am. J. Physiol. Heart Circ. Physiol..

[58] Dimitriadis EK, Horkay F, Maresca J, Kachar B, Chadwick RS (2002). Determination of elastic moduli of thin layers of soft material using the atomic force microscope. Biophys. J..

[59] Gavara N, Chadwick RS (2012). Determination of the elastic moduli of thin samples and adherent cells using conical atomic force microscope tips. Nat. Nanotechnol..

[60] Costa KD, Yin FC (1999). Analysis of indentation: implications for measuring mechanical properties with atomic force microscopy. J. Biomech. Eng..

[61] Azeloglu EU (2014). Interconnected network motifs control podocyte morphology and kidney function. Sci. Signal..

