# Supplementary material for: Disruption of podocyte cytoskeletal biomechanics by dasatinib leads to nephrotoxicity
Source: Nat Commun. 2019 May 3;10:2061. doi: 10.1038/s41467-019-09936-x (PMC6499885; doi:10.1038/s41467-019-09936-x)
Supplement: Supplementary file 1 — Supplementary Info [file 41467_2019_9936_MOESM1_ESM.pdf]

Disruption of podocyte cytoskeletal biomechanics by dasatinib leads to nephrotoxicity

Supplementary Information

Calizo *et al.* 2019

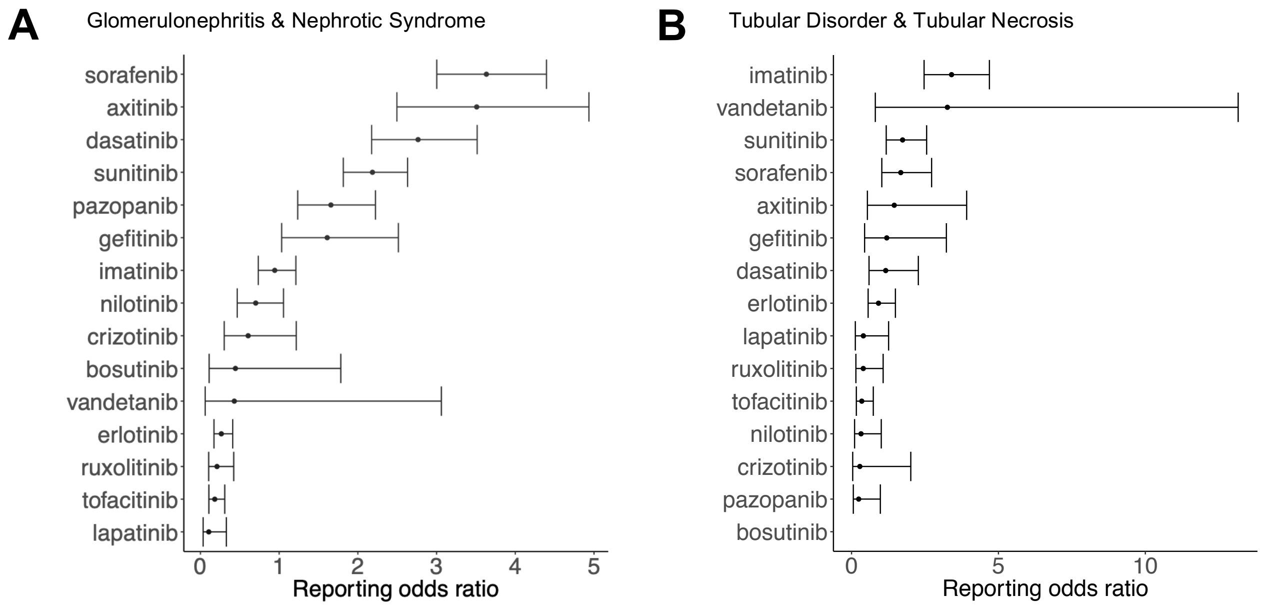

Supplementary Figure 1. Ranking of KI reporting odds ratios (RORs) for adverse event (ADR) subcategories within nephopathies according to the FAERS database (plotted as median value and the range). Compared to its ranking among all nephropathies, dasatinib's relative ranking increased for **(A)** glomerulaonephritis and nephrotic syndrome ROR, but decreased for **(B)** tubular disorder and tubular necrosis RORs.

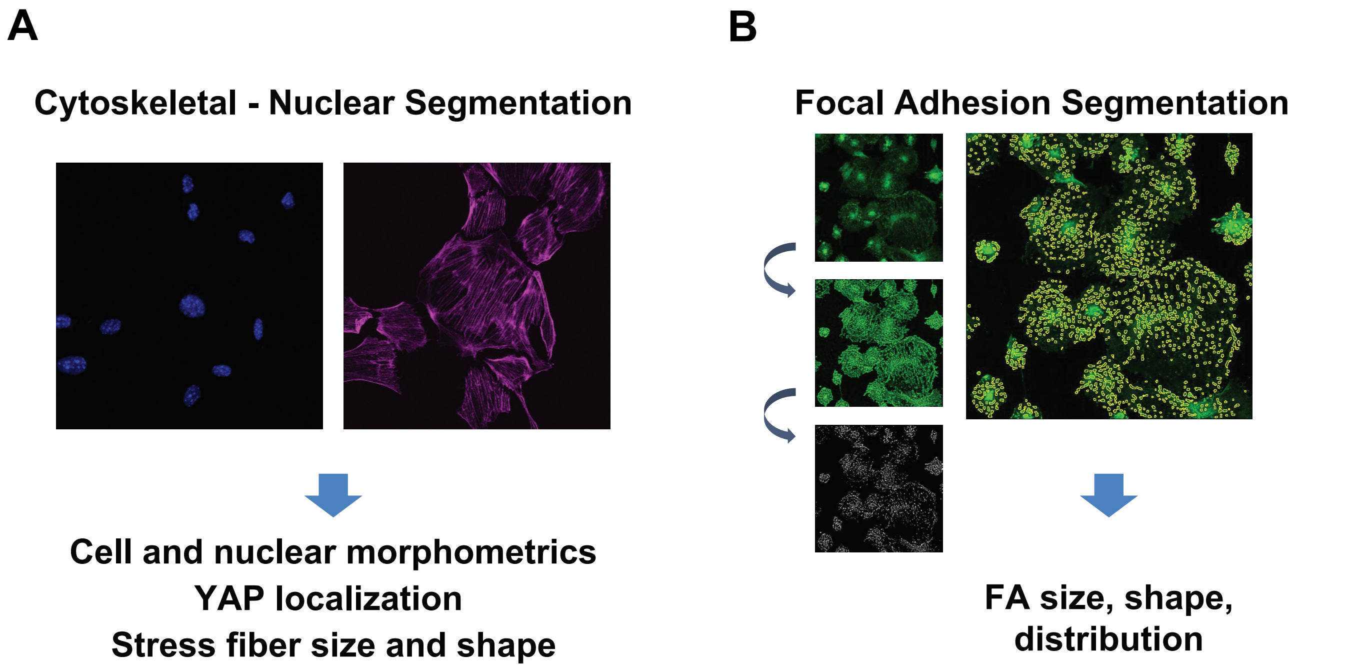

Supplementary Figure 2. Schematic representation of the high-content image analysis (HCA) pipeline that combined open-source Cell Profiler and ImageJ platforms with custom-developed Matlab scripts in order to characterize **(A)** cell, nuclear, and cytoskeletal morphometrics, as well as **(B)** focal adhesion architecture in cultured podocytes treated with kinase inhibitors (KIs). Cell and nuclear morphometrics were performed using images with 200X magnification while stress fiber and focal adhesion metrics were quantified using images with 400X magnification.

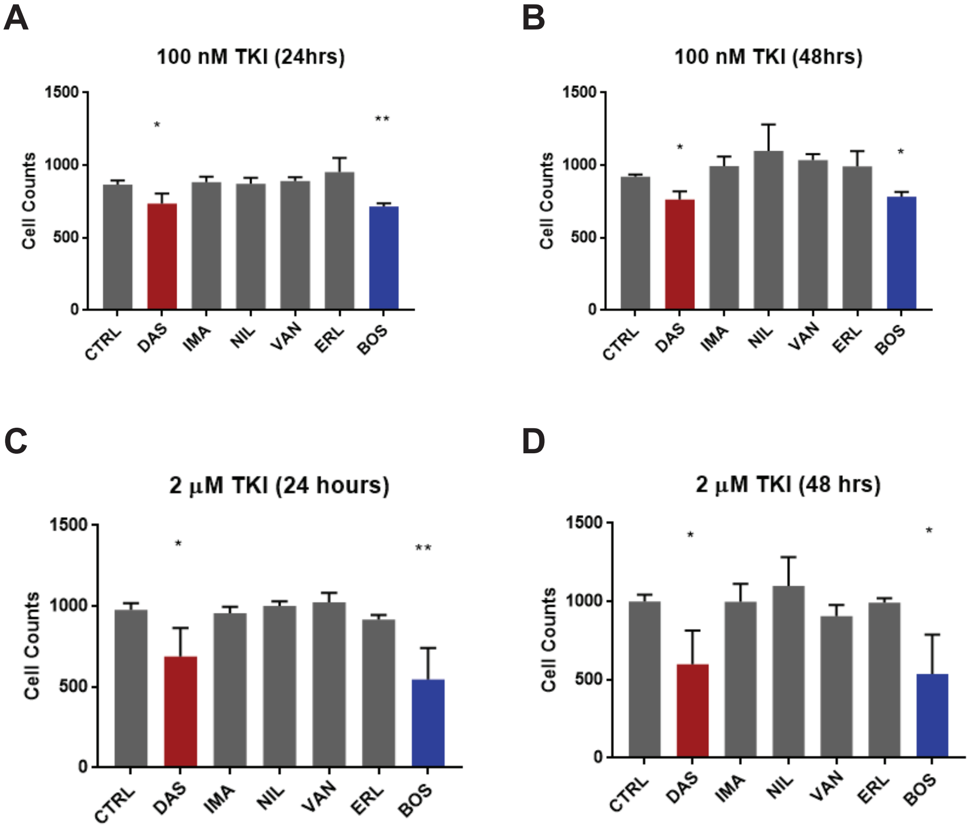

Supplementary Figure 3. Cell viability of cultured podocytes after treatment with different KIs **(A)** at 100 nM for 24 hours, **(B)** at 100 nM for 48 hours, **(C)** at 2 μM for 24 hours, and **(D)** at 2 μM for 48 hours, as assessed by counting of nuclei at the end of treatment culture period. These quantitative immunofluorescence-based assays were performed in addition to the MTT viability assay as secondary validation. In agreement with the MTT assays, only dasatinib and bosutinib had a significant effect on the number of podocytes (mean ± SD; *p < 0.05 or **p < 0.01, Kruskal-Wallis one-way ANOVA followed by Tukey post-hoc multiple comparison).

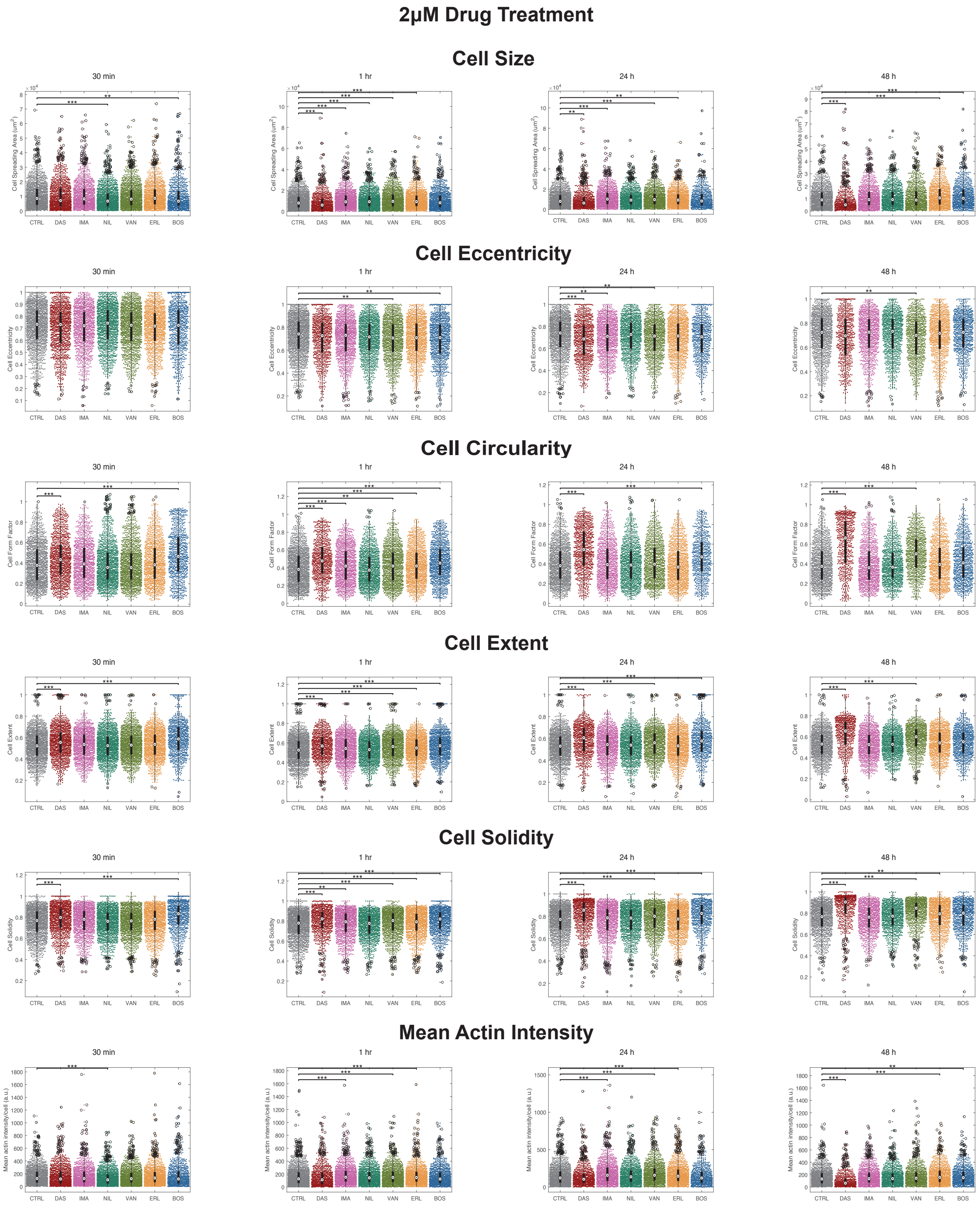

Supplementary Figure 4. Spatiotemporal podocyte cellular morphometrics at 30 minutes, 1 hour, 24 hours and 48 hours after treatment with 2 μM of the selected KIs. CTRL: control, DAS: dasatinib, IMA: imatinib, NIL: nilotinib, VAN: vandetinib, ERL: erlotinib, BOS: bosutinib (***p < 0.001, Kruskal-Wallis one-way ANOVA followed by Tukey post-hoc multiple comparison).

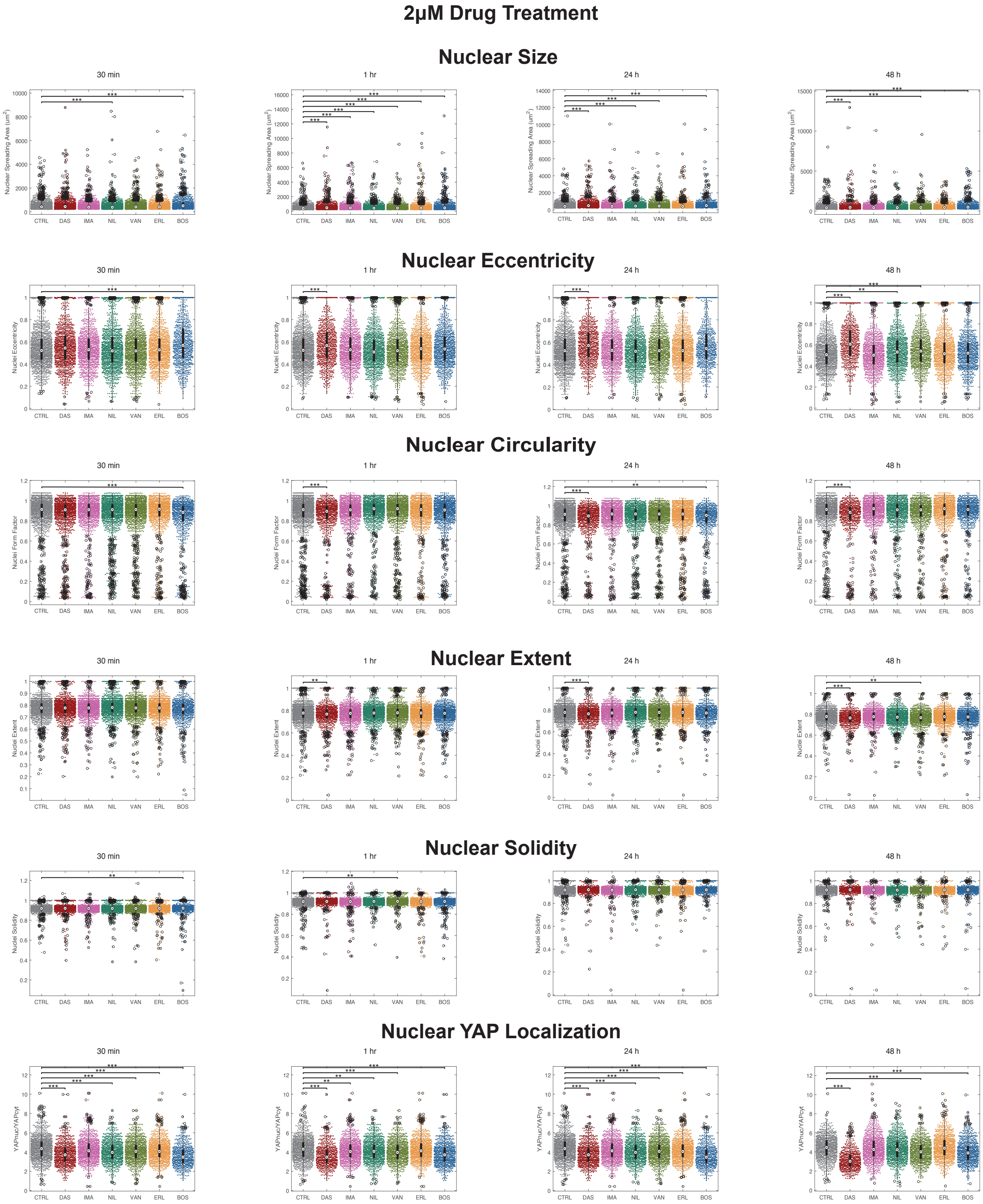

Supplementary Figure 5. Spatiotemporal podocyte nuclear morphometrics at 30 minutes, 1 hour, 24 hours and 48 hours after treatment with 2 µM of the selected KIs. CTRL: control, DAS: dasatinib, IMA: imatinib, NIL: nilotinib, VAN: vandetinib, ERL: erlotinib, BOS: bosutinib (***$p < 0.001$, Kruskal-Wallis one-way ANOVA followed by Tukey post-hoc multiple comparison).

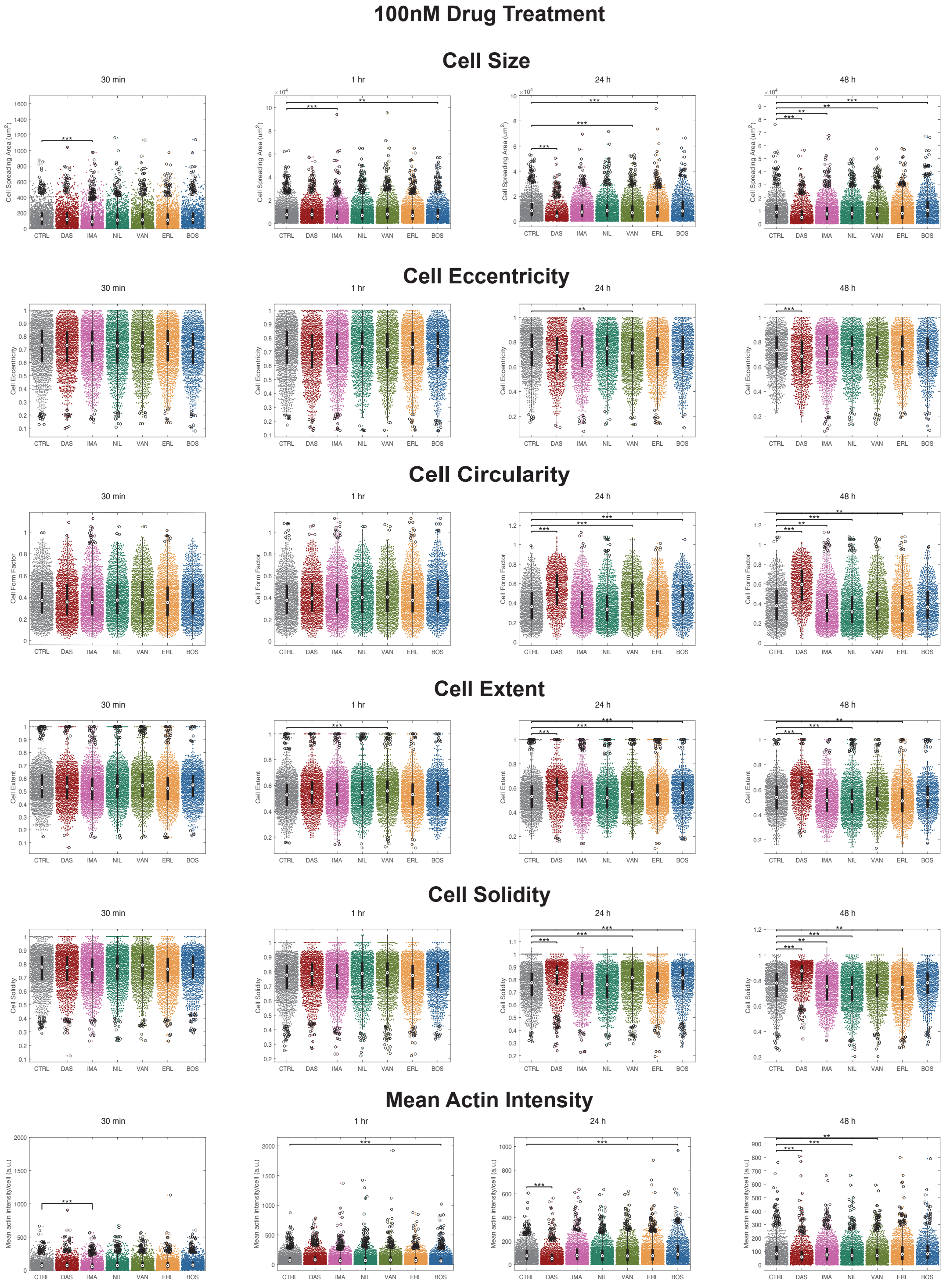

Supplementary Figure 6. Spatiotemporal podocyte <u>cellular</u> morphometrics at 30 minutes, 1 hour, 24 hours and 48 hours after treatment with <u>0.1 μM</u> of the selected KIs. CTRL: control, DAS: dasatinib, IMA: imatinib, NIL: nilotinib, VAN: vandetinib, ERL: erlotinib, BOS: bosutinib (***p < 0.001, Kruskal-Wallis one-way ANOVA followed by Tukey post-hoc multiple comparison).

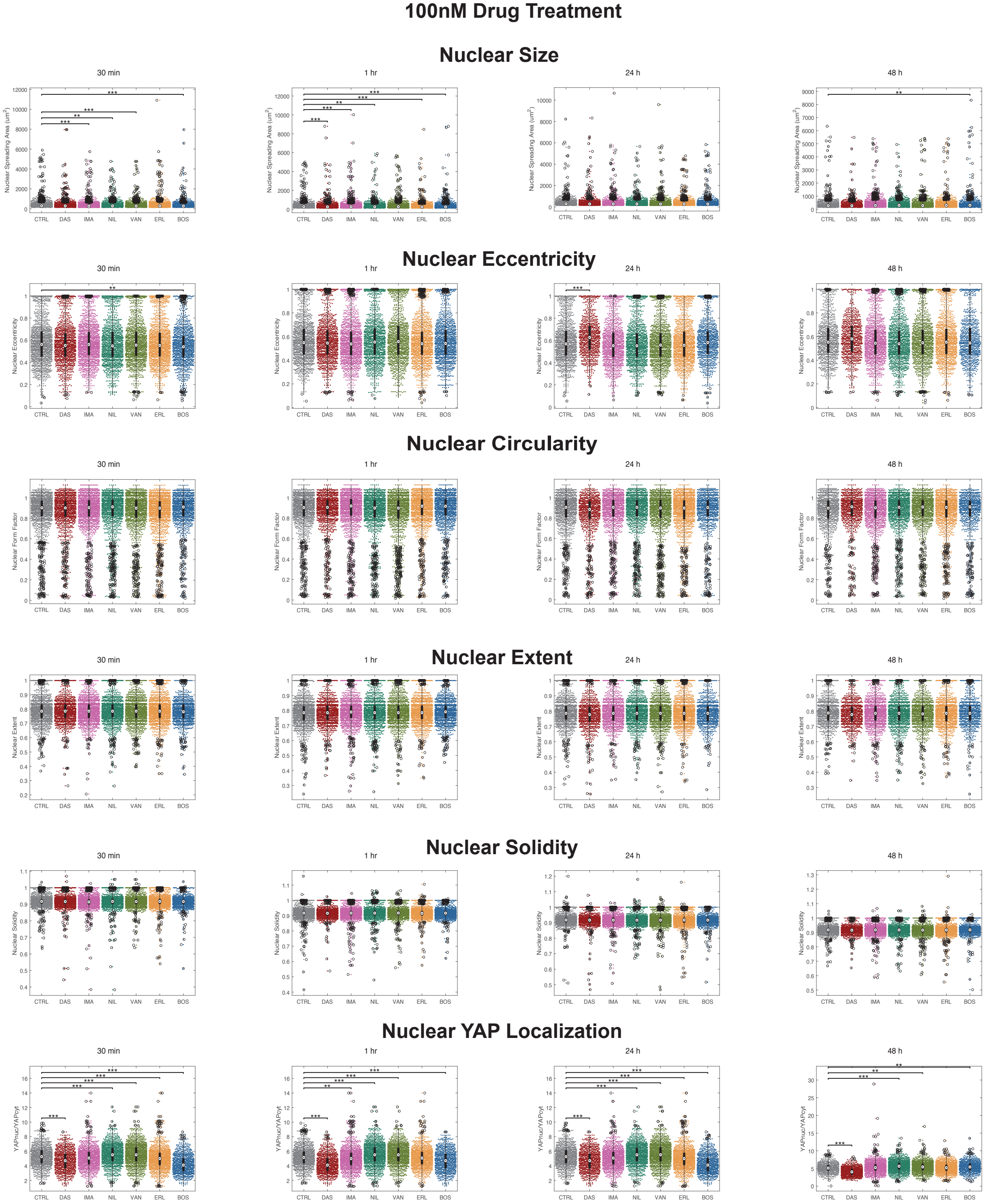

Supplementary Figure 7. Spatiotemporal podocyte nuclear morphometrics at 30 minutes, 1 hour, 24 hours and 48 hours after treatment with 0.1 μM of the selected KIs. CTRL: control, DAS: dasatinib, IMA: imatinib, NIL: nilotinib, VAN: vandetinib, ERL: erlotinib, BOS: bosutinib (***p < 0.001, Kruskal-Wallis one-way ANOVA followed by Tukey post-hoc multiple comparison).

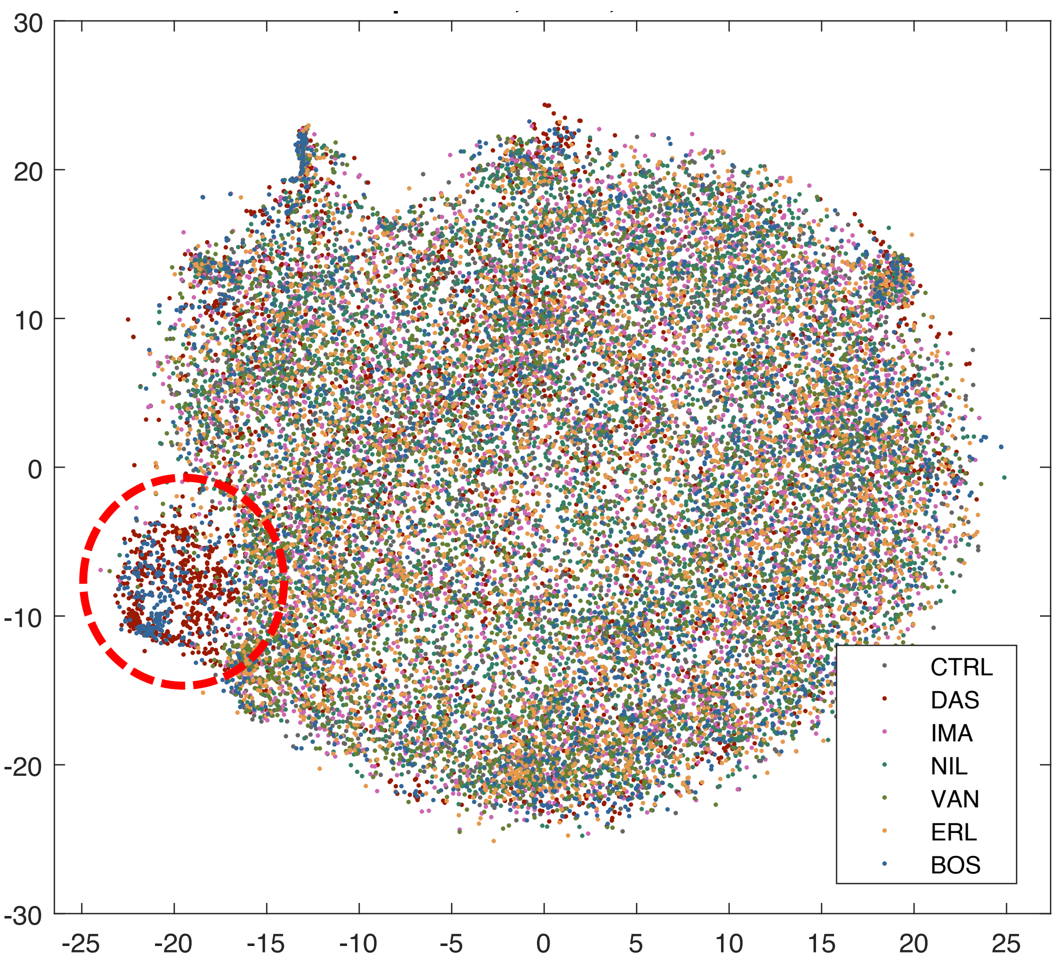

Supplementary Figure 8. Nonlinear dimensional reduction for the 60 morphological and textural characteristics of cultured podocytes using t-distributed stochastic neighbor embedding (tSNE) shows clear clustering of dasatinib treated podocytes (red dashed-circle) while control cells or cells treated with other KIs do not cluster.

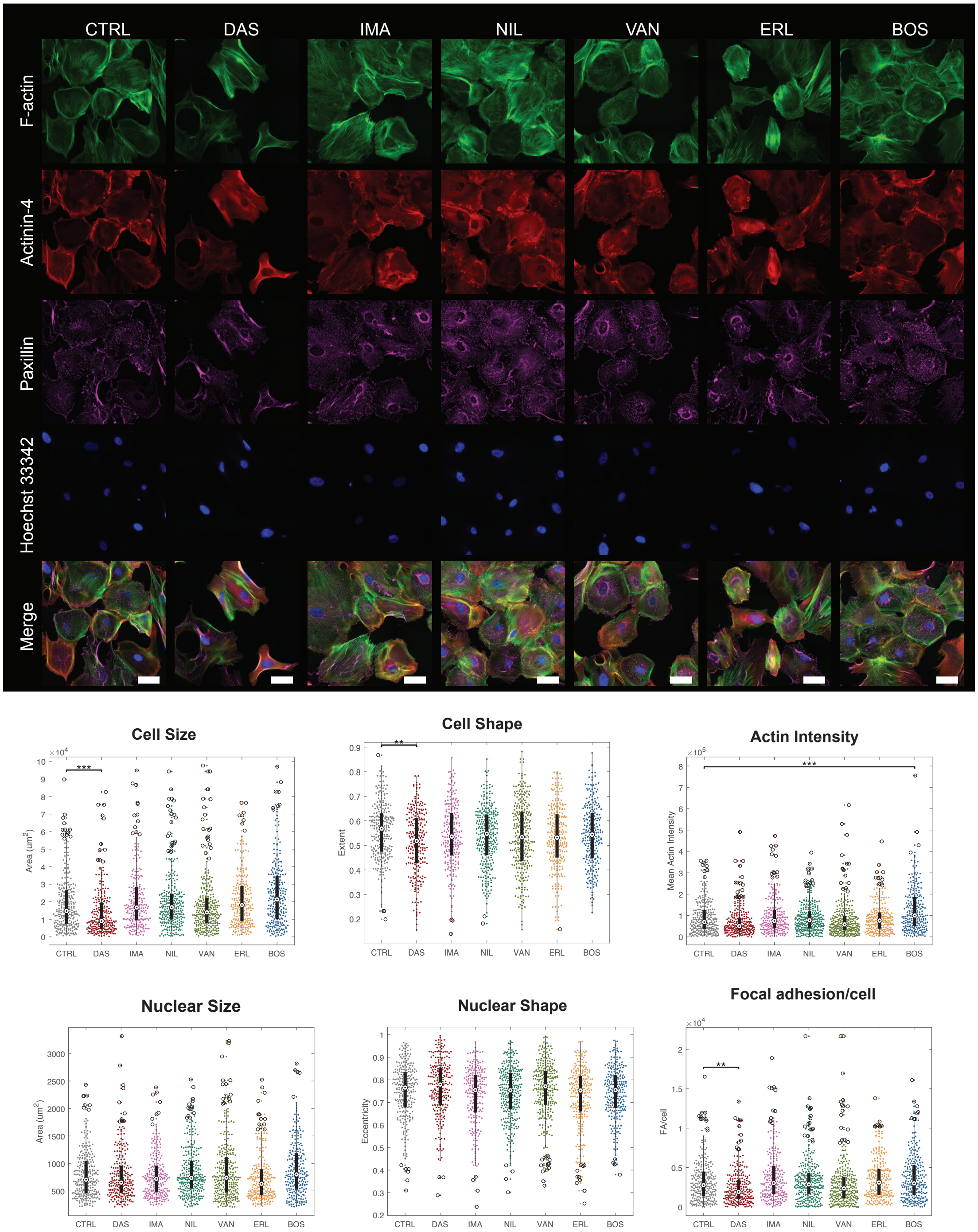

Supplementary Figure 9. Morphometrics for cultured immortalized human podocytes treated with 2 µM of the selected KIs for 24 hours show characteristics similar to immortalized mouse podocytes. CTRL: control, DAS: dasatinib, IMA: imatinib, NIL: nilotinib, VAN: vandetinib, ERL: erlotinib, BOS: bosutinib. Scale bars = 100 µm. (median and the middle quartiles; $^{**}p<0.01$, $^{***}p < 0.001$, Kruskal-Wallis one-way ANOVA followed by post-hoc Tukey multiple comparison)

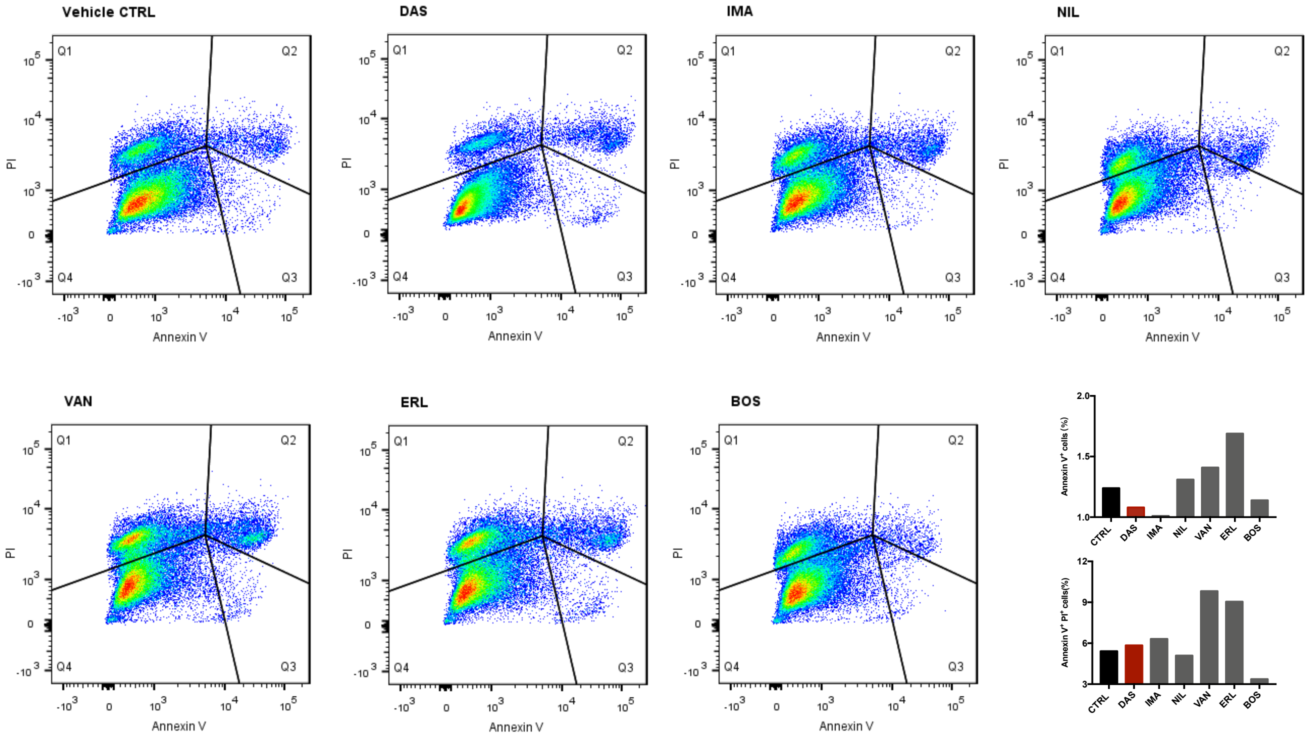

Supplementary Figure 10. Flow cytometry-based Annexin V assay showed similar results to western blot and immunofluorescence-based HCA results whereby several KIs showed slightly increased apoptosis; however, dasatinib did not have a unique effect. Shown gating schema was applied *post hoc* to all conditions identically; no additional preliminary gating was applied.

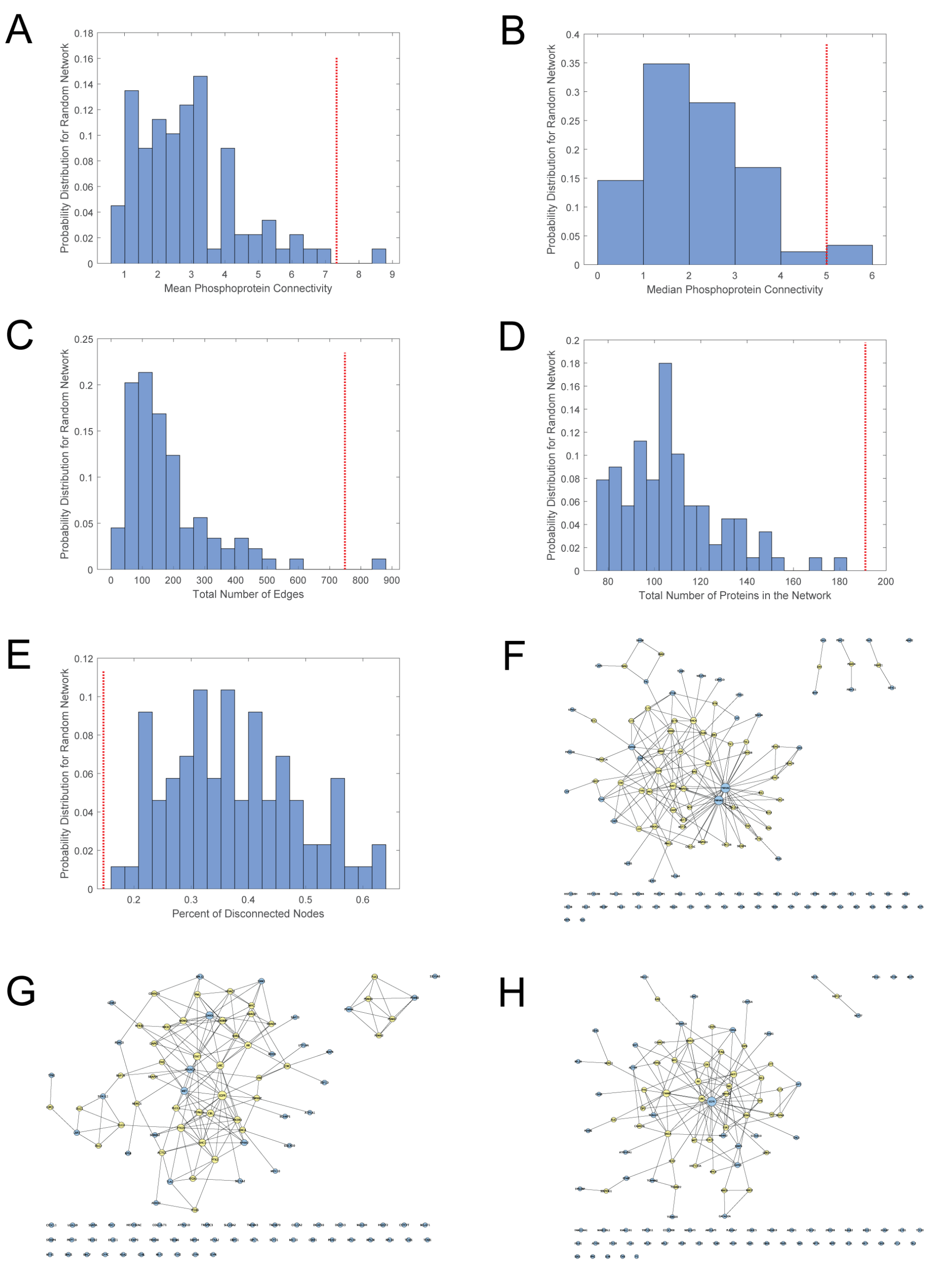

Supplementary Figure 11. Quantitative characterization of connectivity for the protein-protein interaction network from dasatinib treated podocytes shown in Figure 5C. Network was constructed with the nearest neighbors approach using 76 differentially tyrosine-phophorylated proteins shown in Supplementary Table 3 as the seed nodes. To compare connectivity metrics, 200 random networks were generated by selecting 76 random nodes from the same proteomic dataset. Almost all random networks had lower **(A)** mean or **(B)** median per node connectivity and **(C)** lower total number of edges. **(D)** The random networks were always smaller in size, and **(E)** they always had more disconnected nodes than the phospho-proteomically identified network. **(F-H)** Representative random networks exhibit highly discordant behavior compared to the interconnected cohesive network obtained by the differential phospho-proteins in Figure 5C. Node size is proportional to connectivity; cyan = seed nodes, yellow = intermediate nodes drawn from the human protein-protein interactome.

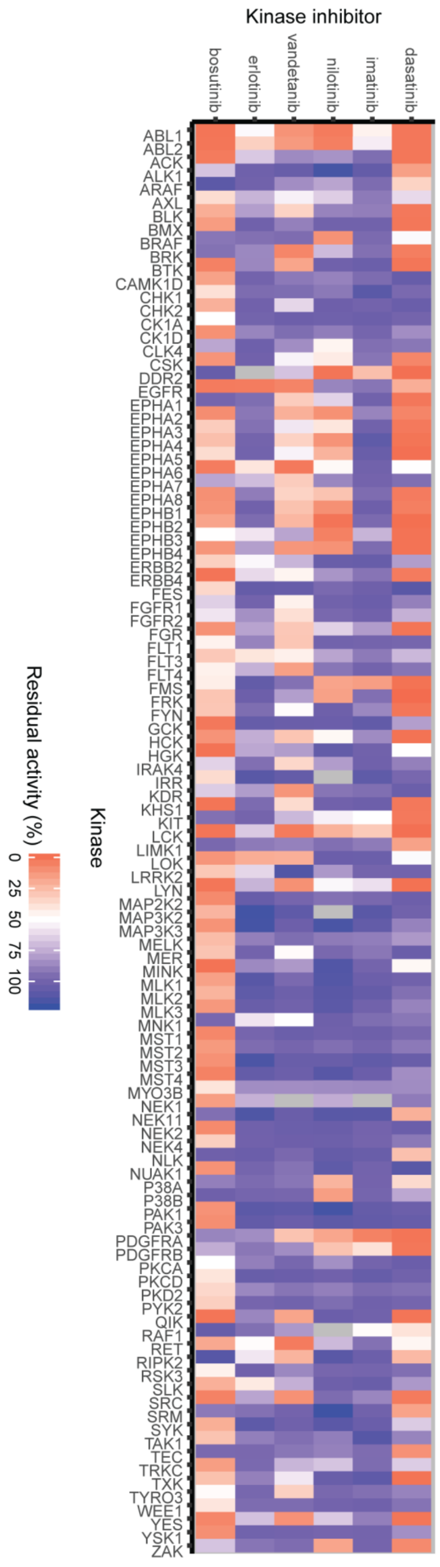

Supplementary Figure 12. Complete unfiltered kinome profiling data, showing the residual activity of kinases that were inhibited by at least one of the six tested KIs.

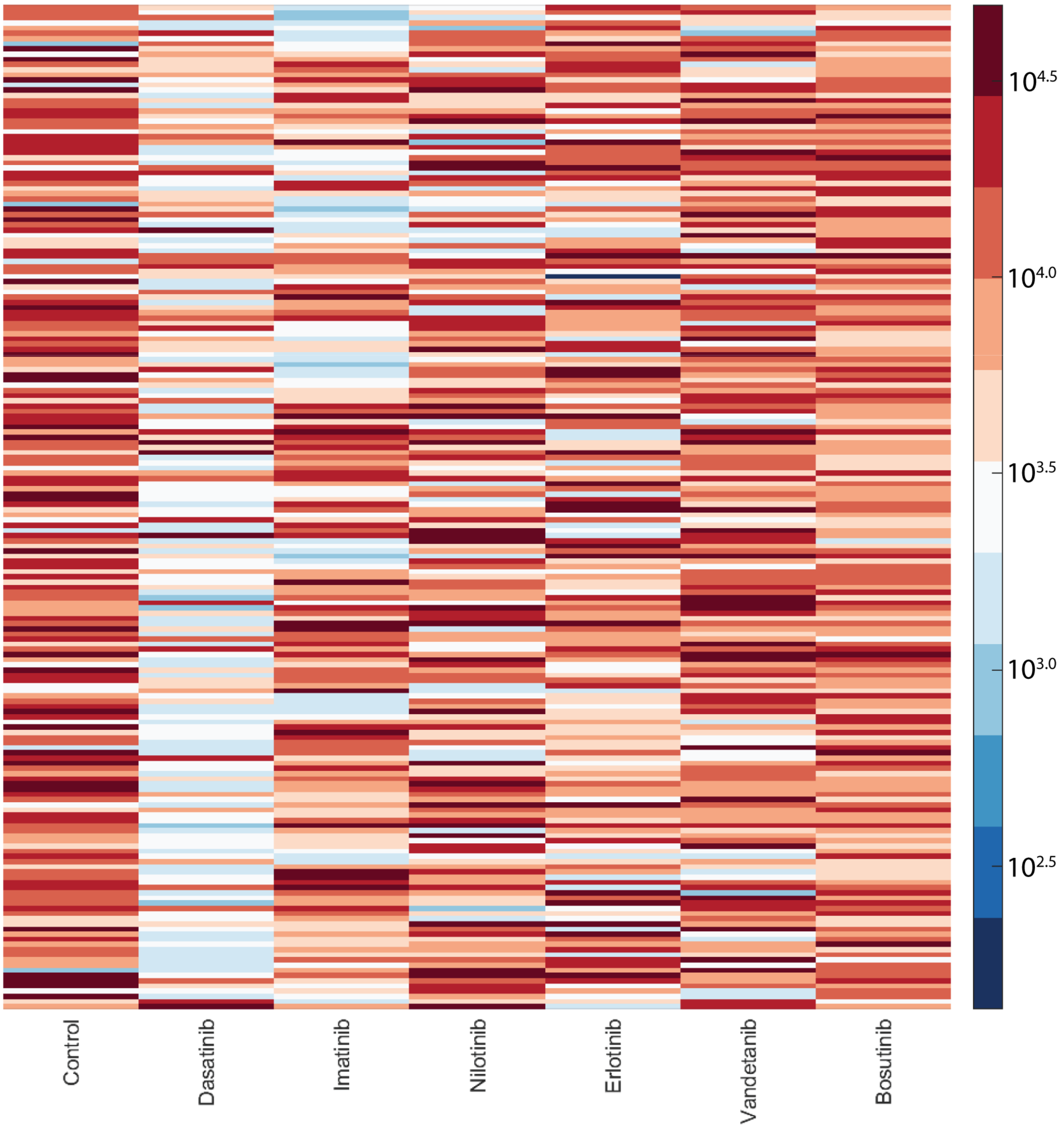

Supplementary Figure 13. Heatmap for atomic force microscope indentations showing the raw log-normalized apparent elastic modulus for kidney podocytes at baseline and under treatment with the selected KIs. Units are shown in kPa.

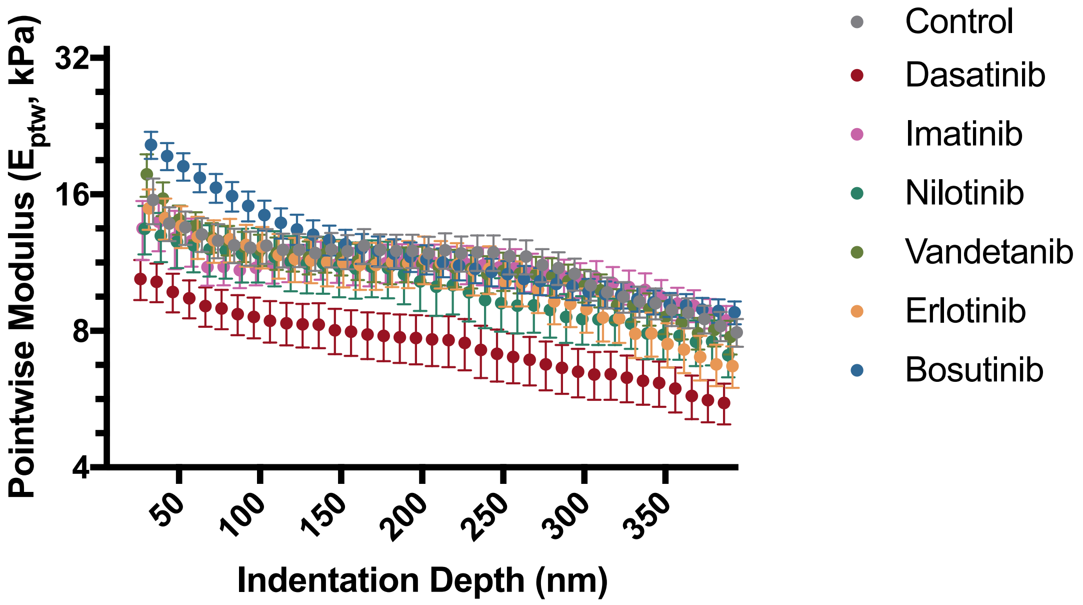

Supplementary Figure 14. Depth-dependent pointwise apparent elastic modulus (E_{ptw}) values for mouse kidney podocytes treated with vehicle control or KIs (mean ± SEM). Pointwise modulus values did not show any increase within the first half micron of indentation suggesting little or no substrate-induced stiffening effect. Furthermore, the asymptotic values observed for pointwise moduli agreed well with the Hertzian-fitted elastic moduli. Two-way ANOVA followed by post-hoc Tukey test showed that only dasatinib was significantly different from control. We also noted that bosutinib had a slight stiffening effect in the initial range of indentation that agreed with the HCA-observed minor increase in actin crosslinking with that drug treatment.

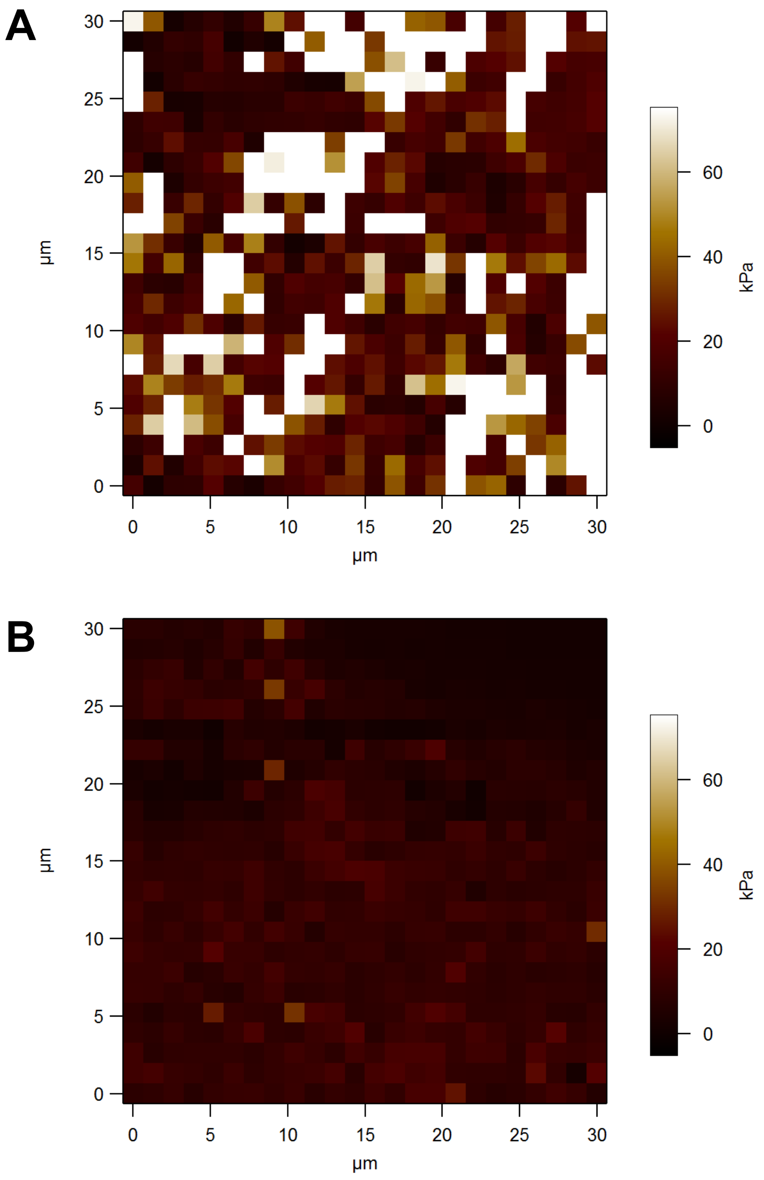

Supplementary Figure 15. Enlarged versions of the AFM elastography maps shown in Figure 6C covering 31 x 31 μm^2 area with 1,024 distinct indentations for **(A)** vehicle-treated control and **(B)** dasatinib-treated podocytes.

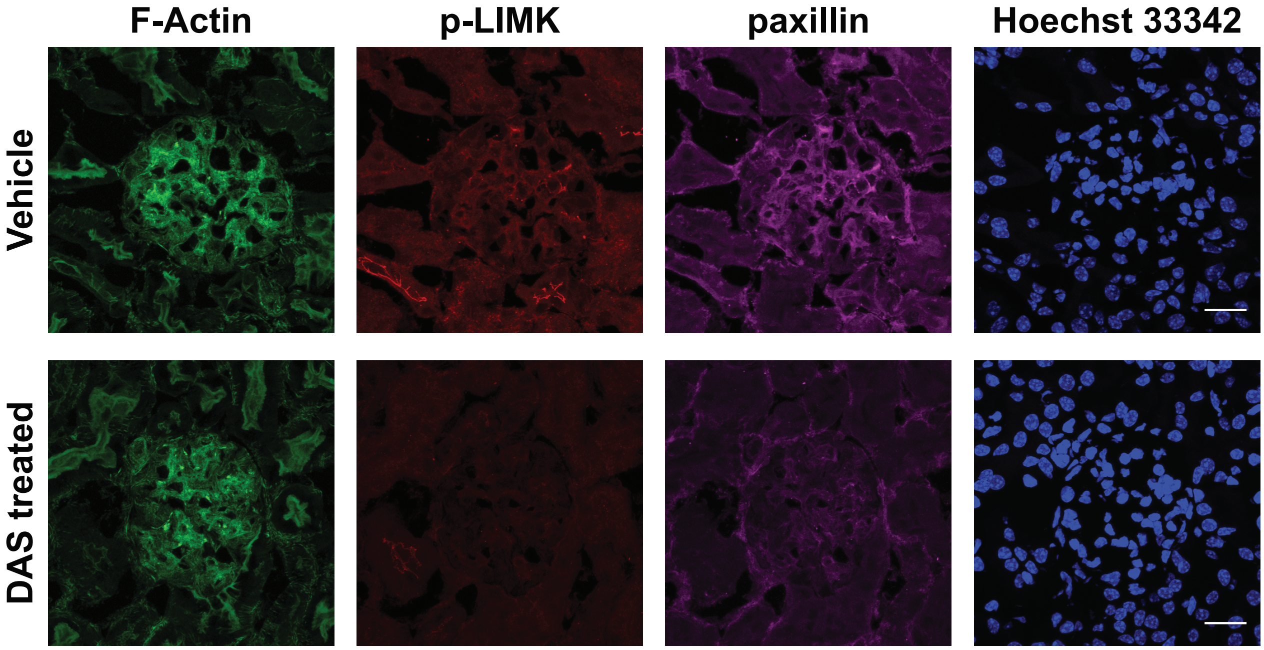

Supplementary Figure 16. Representative immunofluorescence imaging shows decreased phospho-LIM kinase (p-LIMK) and paxillin staining in glomeruli of mice that had been chronically treated with dasatinib for five weeks. Scale bars = 20 μm.

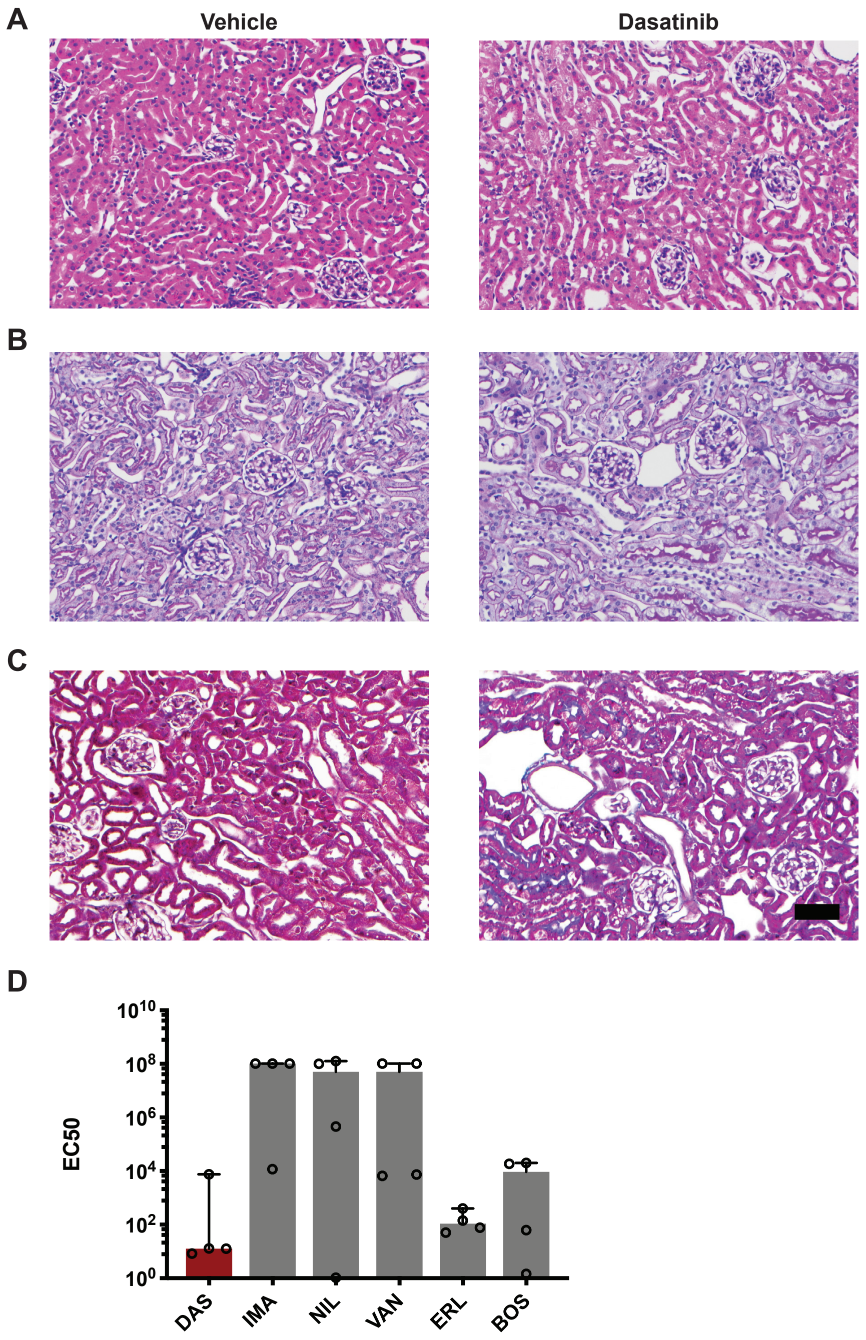

Supplementary Figure 17. *In vivo* histopathological and *in vitro* cytotoxic effects of dasatinib on kidney tubular epithelia were minimal. **(A)** H&E, **(B)** PAS, and **(C)** trichrome staining showed little difference between vehicle and dasatinib treated wild-type 129S1/SvImJ mice (scale bar = 100 μm). **(D)** Dasatinib exhibited similar cytotoxicity towards tubular epithelial cells and podocytes. However, unlike podocytes, dasatinib EC50 values against tubular epithelial cells were very similar with erlotinib, which has a peak plasma concentration that is 50X higher than that of dasatinib (geometric mean ± geometric SD, not significant, Kruskal-Wallis one-way ANOVA).

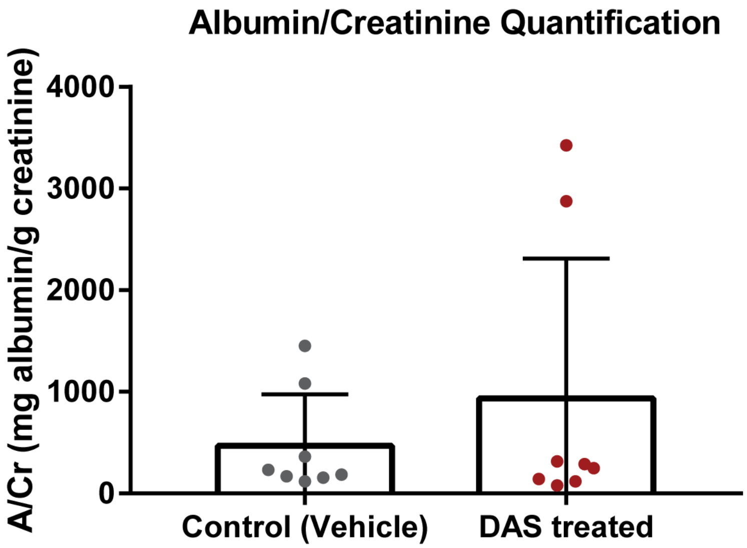

Supplementary Figure 18. Urine albumin/creatinine ratio of wild-type 129S1/SvImJ mice treated daily with oral dasatinib (or vehicle control) for five weeks (mean ± SEM; not significant, unpaired t-test).

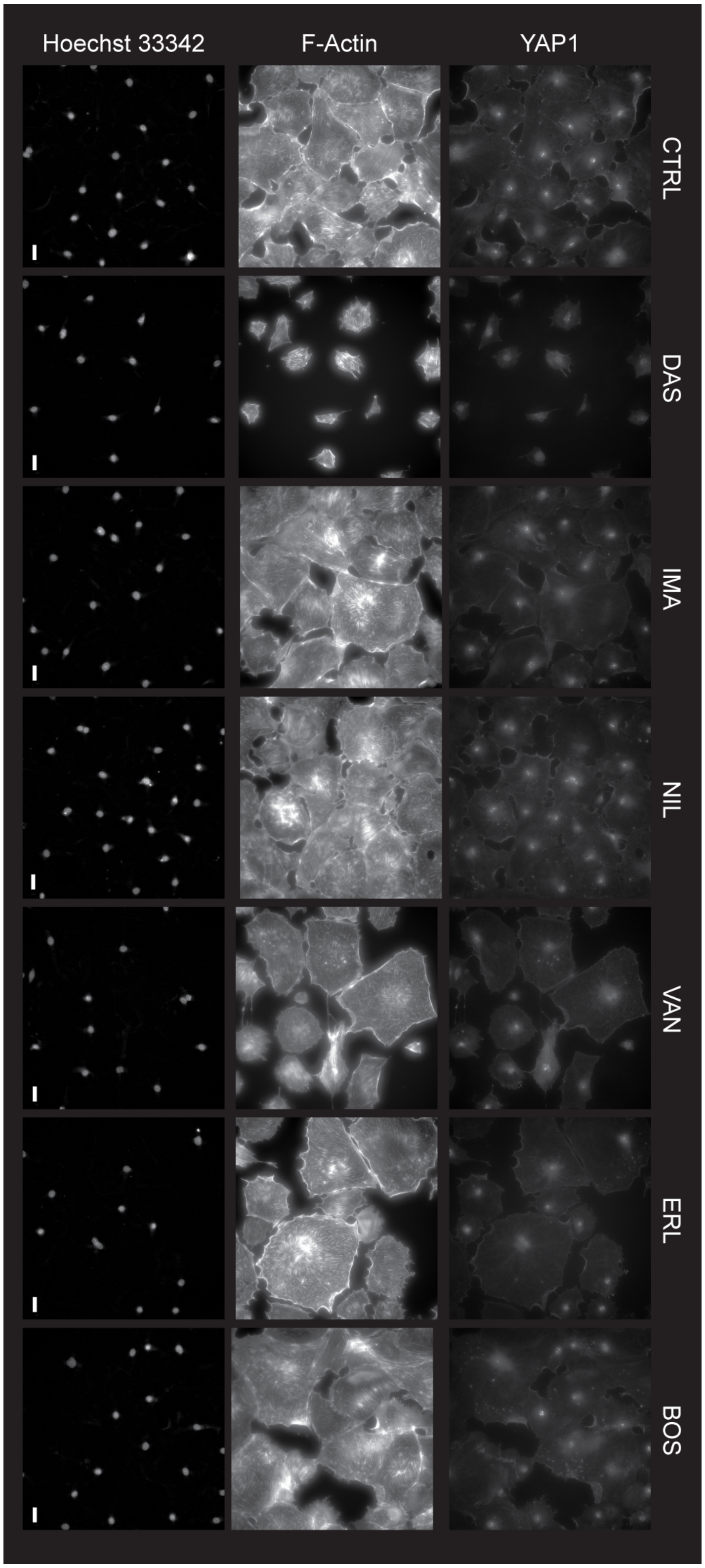

Supplementary Figure 19. Representative images showing subcellular localization of YAP in immortalized mouse kidney podocytes treated with vehicle control and other KIs for 24 hours. Nuclear YAP localization was significantly and consistently reduced in dasatinib group. Scale bars = 10 µm.

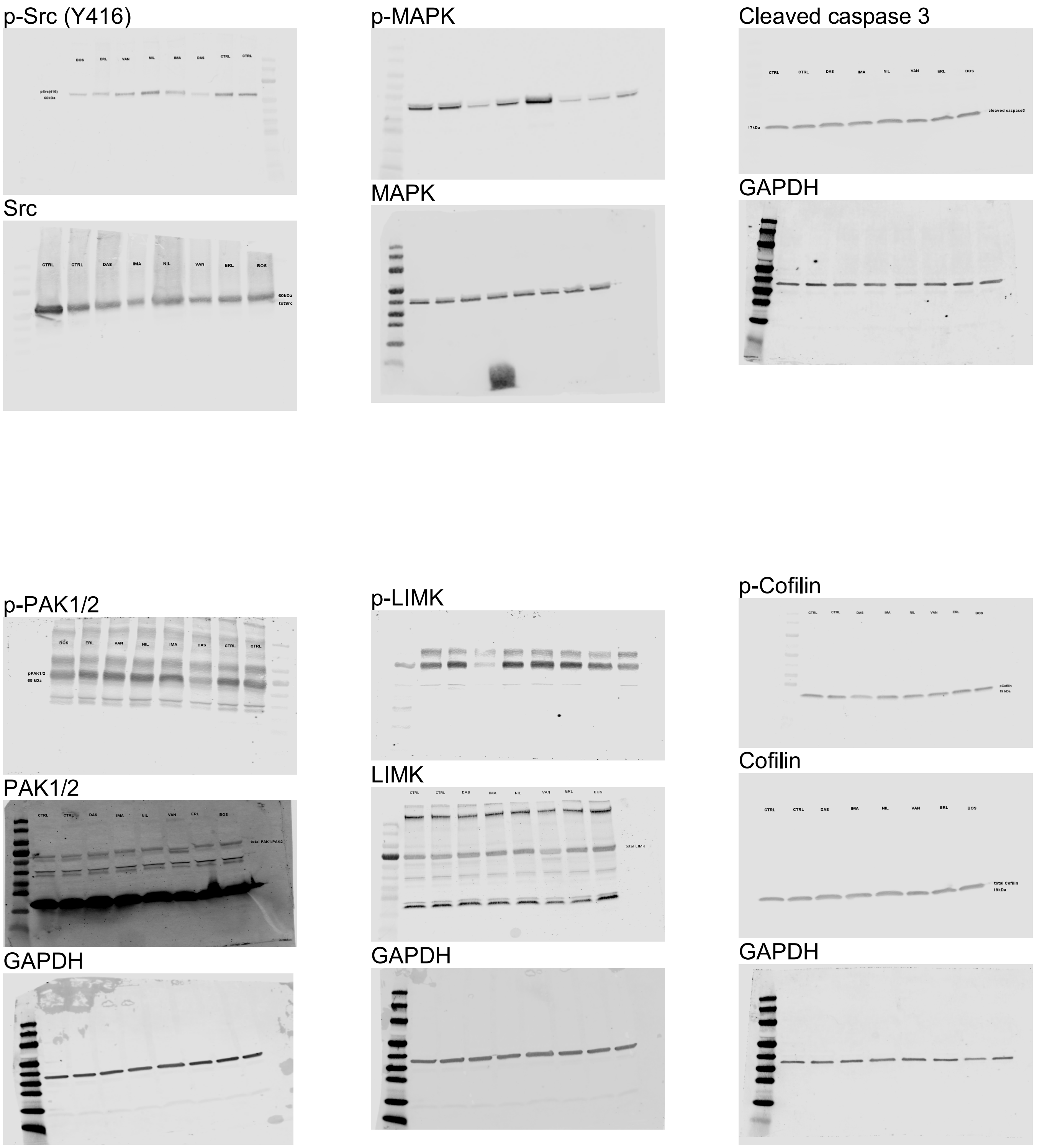

Supplementary Figure 20. Uncropped scans of all western blots presented in the paper. Samples were loaded in the same order presented throughout the paper: CTRL-CTRL-DAS-IMA-NIL-VAN-ERL-BOS, and resolved on 4-20% gradient gels (Bio-Rad). Imaging was performed at 16-bit-depth using the fluorescence-based Li-Cor CLx digital laser scanning system. The protein ladder used was PageRuler Plus (Thermo Fisher) with sizes: 250 – 130 – 100 – 70 – 55 – 35 – 25 –15 – 10 kDa.

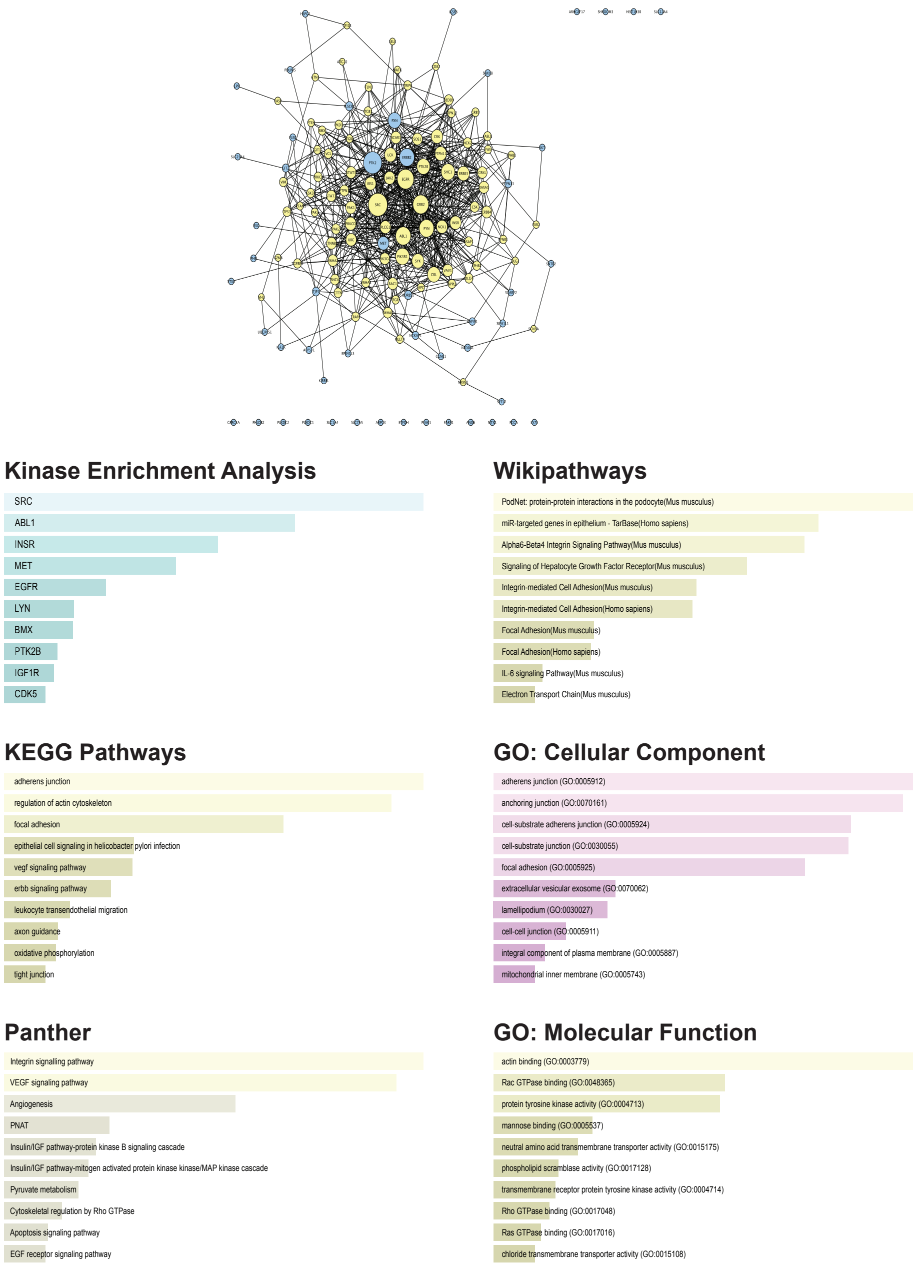

Supplementary Figure 21. Biological replicate for the phospho-proteomics experiment shown in Figure 5. Differentially expressed proteins according in phospho-tyrosine enriched shotgun proteomics were used to construct a protein-protein interaction network, which produced similar results to the first experiment with focal adhesion and actin-associated proteins being significantly over-represented. Enrichment analysis showed similar results with actin and focal adhesion related processes ranking high in all enriched pathway and ontology group.

Supplementary Table 1. Gene and protein acronyms

Protein Name	Full Name	Synonym(s)
**ABL1**	Abelson tyrosine-protein kinase 1	ABLL, ARG
**ABL2**	Abelson tyrosine-protein kinase 2	ABLL, ARG
**BCR**	Breakpoint cluster region protein	BCR1
**BRAF**	Serine/threonine-protein kinase B-raf	BRAF1
**CC3**	Cleaved caspase 3	CASP3
**EGFR**	Epidermal growth factor (EGF) receptor	ERBB1, HER1
**EPHA3**	EPH receptor A3	ETK, TYRO4
**EPHA5**	EPH receptor A5	EHK, TYRO4
**FES**	Proto-oncogene c-Fes/Fps	FES
**HCK**	Hemopoietic cell kinase	HCK
**LCK**	Leukocyte C-terminal Src kinase	LSK
**LIMK1**	LIM kinase	LIMK, LIMK-1
**LRRK2**	Leucine-rich repeat kinase 2	DARDARIN
**LYN**	Lck/Yes-related novel tyrosine kinase	JTK8
**MAPK1/2**	Mitogen-activated kinase 1 and 2	ERK2/1
**MTOR**	Mammalian target of rapamycin	FRAP1
**PAK1**	p21-activated kinase 1	PAK-1
**PAK3**	p21-activated kinase 3	PAK-3
**PDGFRA**	Platelet-derived growth factor receptor α	PDGFR-2, CD140a
**PDGFRB**	Platelet-derived growth factor receptor β	PDGFR-1, CD140b
**PDL1**	Programmed cell death 1 ligand 1	CD274, B7-H1
**RANKL**	Receptor activator of nuclear factor kappa-B ligand	TNFSF11, CD254
**SRC**	Proto-oncogene tyrosine kinase c-Src	c-SRC
**SYNPO**	Synaptopodin	
**VEGF**	Vascular endothelial growth factor	VEGFA
**WT1**	Wilms tumor 1	WT-1

Supplementary Table 2. Key metrics for the KIs selected from the FAERS database ranked according to their nephrotoxicity risk

Drug	Trade Name	Odds Ratio	Plasma C_{max}	Intended Target	Common Use
**Dasatinib**	Sprycell	1.34-2.01	37 ng/mL	BCR-ABL1, SRC	Chronic myeloid leukemia (CML)
**Imatinib**	Gleevec	1.01-1.36	1822 ng/ml	BCR-ABL1	CML
**Nilotinib**	Tasigna	0.50-0.89	1644 ng/ml	BCR-ABL1	CML
**Vandetanib**	Caprelsa	0.19-1.79	178 ng/ml	VEGFR, RET	Medullary thyroid cancer
**Erlotinib**	Tarceva	0.32-0.55	1140 ng/ml	EGFR	Non-small cell lung cancer
**Bosutinib**	Bosulif	0.12-1.24	34 ng/ml	BCR-ABL1, SRC	CML

Supplementary Table 3. Proteins significantly downregulated according to phospho-tyrosine enriched shotgun proteomics as shown in Figure 5A

Protein Name	Gene ID	Uniprot ID	Mass	Vehicle Spectra			Dasatinib Spectra			log2FC
116 kDa U5 small nuclear ribonucleoprotein component	Eftud2	O08810	109 kDa	22	21	13	10	5	8	-1.3
40S ribosomal protein S4, X isoform	Rps4x	P62702	30 kDa	12	10	9	8	7	8	-0.4
60S ribosomal protein L4	Rpl4	Q9D8E6	47 kDa	18	20	16	15	9	10	-0.7
Alpha-actinin-4	Actn4	P57780	105 kDa	214	235	241	205	189	186	-0.3
AP-2 complex subunit alpha-1	Ap2a1	P17426	108 kDa	35	34	28	22	17	17	-0.8
AP-2 complex subunit beta	Ap2b1	Q9DBG3	105 kDa	55	52	44	37	30	27	-0.7
ARF GTPase-activating protein GIT1	Git1	Q68FF6	85 kDa	7	9	8	2	3	2	-1.8
Breast cancer anti-estrogen resistance protein-1	Bcar1	Q61140	94 kDa	12	15	11	9	4	7	-0.9
Calcium homeostasis endoplasmic reticulum protein	Cherp	Q8CGZ0	106 kDa	5	4	4	1	2	2	-1.4
Cell cycle control protein 50A	Tmem30a	Q8VEK0	41 kDa	4	6	3	2	1	2	-1.4
Clathrin heavy chain 1	Cltc	Q68FD5	192 kDa	53	46	36	28	26	24	-0.8
Core histone macro-H2A.1	H2afy	Q9QZQ8	40 kDa	68	65	63	59	62	61	-0.1
Dephospho-CoA kinase domain-containing protein	Dcakd	Q8BHC4	26 kDa	4	4	3	2	2	2	-0.9
Drebrin	Dbn1	Q9QXS6	77 kDa	31	32	36	23	27	28	-0.3
Endoplasmic reticulum-Golgi intermediate compartment protein-1	Ergic1	Q9DC16	33 kDa	14	14	16	13	12	10	-0.3
Enhancer of mRNA-decapping protein 4	Edc4	Q3UJB9	152 kDa	7	5	4	2	2	2	-1.4
Epidermal growth factor receptor substrate 15-like-1	Eps15l1	Q60902	99 kDa	7	4	6	4	2	2	-1.1
Extended synaptotagmin-1	Esyt1	Q3U7R1	122 kDa	25	25	17	13	11	9	-1.0
Fibulin-2	Fbln2	P37889	132 kDa	101	95	88	86	79	84	-0.2
Filamin-C	Flnc	Q8VHX6	291 kDa	53	47	42	40	31	26	-0.5
Focal adhesion kinase-1	Ptk2	P34152	124 kDa	5	6	4	2	1	1	-1.9
Golgin subfamily A member-2	Golga2	Q921M4	113 kDa	5	5	5	2	1	2	-1.6
Heterogeneous nuclear ribonucleoprotein D0	Hnrnpd	Q60668	38 kDa	5	6	7	2	3	4	-1.0
Heterogeneous nuclear ribonucleoprotein U-like protein-2	Hnrnpul2	Q00PI9	85 kDa	9	13	10	5	6	6	-0.9
Histone deacetylase 1	Hdac1	O09106	55 kDa	6	5	5	2	2	4	-1.0
Inositol monophosphatase-3	Impad1	Q80V26	39 kDa	4	5	4	1	2	3	-1.1
Integrin alpha-V	Itgav	P43406	115 kDa	6	5	4	3	3	2	-0.9
Kin of IRRE-like protein-1	Kirrel	Q80W68	87 kDa	10	9	7	7	3	3	-1.0
Kinectin	Ktn1	Q61595	153 kDa	23	26	20	14	12	12	-0.9
Liprin-beta-1	Ppfibp1	Q8C8U0	109 kDa	6	4	5	3	1	1	-1.6
Mitochondrial antiviral-signaling protein	Mavs	Q8VCF0	53 kDa	20	21	21	15	16	13	-0.5
Mitochondrial carnitine/acylcarnitine carrier protein	Slc25a20	Q9Z2Z6	33 kDa	7	5	5	4	2	3	-0.9
Myb-binding protein-1A	Mybbp1a	Q7TPV4	152 kDa	11	10	5	2	2	2	-2.1
Myoferlin	Myof	Q69ZN7	233 kDa	58	53	40	27	30	26	-0.9
Myosin light chain kinase, smooth muscle	Mylk	Q6PDN3	213 kDa	20	23	15	10	11	6	-1.1
Myosin phosphatase Rho-interacting protein	Mprip	P97434	116 kDa	31	33	25	11	15	10	-1.3
Myosin-10	Myh10	Q61879	229 kDa	48	36	35	19	19	19	-1.1
Myosin-9	Myh9	Q8VDD5	226 kDa	41	33	39	26	17	17	-0.9
NADH-ubiquinone oxidoreductase 75 kDa subunit, mitochondrial	Ndufs1	Q91VD9	80 kDa	30	33	30	24	25	21	-0.4
Nesprin-3	Syne3	Q4FZC9	112 kDa	7	8	7	2	3	3	-1.5
Neurabin-2	Ppp1r9b	Q6R891	90 kDa	19	22	14	12	11	11	-0.7
Nidogen-1	Nid1	P10493	137 kDa	13	11	8	5	4	5	-1.2
Nidogen-2	Nid2	O88322	154 kDa	80	65	69	60	58	49	-0.4
Nuclease-sensitive element-binding protein-1	Ybx1	P62960	36 kDa	19	22	17	16	13	10	-0.6
Nucleolar transcription factor-1	Ubtf	P25976	90 kDa	6	5	5	4	3	1	-1.0
Nucleolin	Ncl	P09405	77 kDa	9	9	9	6	6	5	-0.7
Paxillin	Pxn	Q8VI36	64 kDa	18	18	20	4	3	3	-2.5
Plectin	Plec	Q9QXS1	534 kDa	264	226	188	173	162	160	-0.5
Poly(rC)-binding protein-2	Pcbp2	Q61990	38 kDa	7	8	7	6	4	3	-0.8
Polymerase I and transcript release factor	Ptrf	O54724	44 kDa	90	89	94	88	85	83	-0.1

Polypyrimidine tract-binding protein-3	Ptbp3	Q8BHD7	57 kDa	5	5	4	1	2	2	-1.5
Procollagen C-endopeptidase enhancer-1	Pcolce	Q61398	50 kDa	8	7	5	4	4	3	-0.9
Protein mago nashi homolog	Magoh	P61327	17 kDa	11	11	9	6	7	9	-0.5
PTB domain-containing engulfment adapter protein-1	Gulp1	Q8K2A1	34 kDa	5	7	4	2	3	3	-1.0
Ras-related protein Rab-5C	Rab5c	P35278	23 kDa	9	8	8	7	7	7	-0.3
Regulation of nuclear pre-mRNA domain-containing protein 1B	Rprd1b	Q9CSU0	37 kDa	4	3	4	3	2	2	-0.7
Reticulon-4	Rtn4	Q99P72	127 kDa	13	14	10	9	8	7	-0.6
Ribosome-binding protein 1	Rrbp1	Q99PL5	173 kDa	45	41	27	24	21	18	-0.8
Sarcoplasmic/endoplasmic reticulum calcium ATPase 2	Atp2a2	O55143	115 kDa	11	9	6	3	5	5	-1.0
Splicing factor 3A subunit-1	Sf3a1	Q8K4Z5	89 kDa	9	6	6	3	3	1	-1.6
Splicing factor, proline- and glutamine-rich	Sfpq	Q8VIJ6	75 kDa	22	17	18	13	8	12	-0.8
Striatin-3	Strn3	Q9ERG2	87 kDa	3	3	4	2	1	1	-1.3
Synemin	Synm	Q70IV5	173 kDa	36	36	29	27	22	22	-0.5
T-complex protein-1 subunit theta	Cct8	P42932	60 kDa	7	6	7	5	5	5	-0.4
TAR DNA-binding protein-43	Tardbp	Q921F2	45 kDa	10	15	13	8	9	8	-0.6
Thyroid hormone receptor-associated protein-3	Thrap3	Q569Z6	108 kDa	14	12	13	10	8	9	-0.5
Tight junction protein ZO-1	Tjp1	P39447	195 kDa	15	19	14	6	10	9	-0.9
TraB domain-containing protein	Trabd	Q99JY4	42 kDa	4	5	5	2	3	1	-1.2
Translocation protein SEC63 homolog	Sec63	Q8VHE0	88 kDa	9	7	5	2	2	2	-1.8
U3 small nucleolar RNA-associated protein 18 homolog	Utp18	Q5SSI6	61 kDa	3	3	2	1	2	1	-1.0
UDP-glucuronic acid decarboxylase-1	Uxs1	Q91XL3	48 kDa	6	7	8	5	4	3	-0.8
Ufm1-specific protease-2	Ufsp2	Q99K23	53 kDa	8	7	7	6	5	4	-0.6
Unconventional myosin-Ic	Myo1c	Q9WTI7	122 kDa	28	26	27	19	18	16	-0.6
V-type proton ATPase subunit B, brain isoform	Atp6v1b2	P62814	57 kDa	26	19	18	13	13	13	-0.7
Vinculin	Vcl	Q64727	117 kDa	70	52	44	28	25	24	-1.1
Zinc finger protein-like-1	Zfpl1	Q9DB43	34 kDa	3	4	3	2	2	2	-0.7

* log2FC = log_2 fold change

Supplementary Table 4. Gene IDs of the protein-protein interaction network impacted by dasatinib and the number of connections for each of the identified nodes

Gene ID	Connectivity
ABL1	21
ACTB	13
ACTC	3
ACTG1	15
ACTN1	8
ACTN4	10
ADRA1B	2
AKT1	20
AMPH	4
AP2A1	15
AP2B1	13
AP2S1	2
AR	17
ARRB1	12
ARRB2	9
ASAP1	5
ATM	4
ATP2A2	2
ATP6V1B2	1
BCAR1	21
BIN1	6
BUB1	3
BUB1B	2
CALM1	12
CAMK2A	11
CAMK2G	6
CCT8	1
CD47	4
CDH1	13
CHD3	3
CHERP	0
CLTC	15
COL4A1	3
CRK	17
CRKL	12
CSK	14
CSNK2A2	10
CTNNA1	7
CTNNB1	23
CTTN	11
CXCR4	5
DBN1	1
DCAKD	0
EDC4	0
EFTUD2	1
EGFR	29
ELN	2
ENTH	2
EPN1	6
EPS15	9
EPS15L1	2
ERGIC1	0
ESR1	17
ESYT1	0
FBLN2	6
FLNC	3
FYN	23
GIT1	11
GIT2	4

Gene ID	Connectivity
PAK1	15
PAK3	5
PBX1	3
PCBP2	3
PCNA	6
PCOLCE	0
PIK3R1	18
PITX2	4
PKD1	10
PLCG1	17
PLEC	2
PPFIA1	4
PPFIA2	5
PPFIA3	4
PPFIBP1	3
PPP1CA	9
PPP1CB	4
PPP1CC	5
PPP1R9B	8
PRKACA	14
PTBP1	4
PTEN	8
PTK2	41
PTK2B	24
PTPN1	12
PTPN11	16
PTPN12	10
PTPN6	13
PTPRF	10
PTPRH	3
PTRF	0
PXN	30
RAB5C	0
RAC1	13
RB1	10
REPS2	3
RHOA	6
RIPK1	3
RPL4	2
RPRD1B	0
RPS4X	2
RRBP1	0
RTN4	0
SEC63	0
SELE	2
SF3A1	3
SFPQ	6
SHC1	22
SIN3A	5
SLC25A20	0
SLC2A4	4
SMAD3	20
SNAP91	4
SNCA	19
SORBS1	3
SP1	10
SPTAN1	5
STAT1	10
STRN3	2

**GJA1**	4
**GOLGA2**	0
**GRIN1**	15
**GSK3B**	17
**GSN**	11
**GULP1**	0
**H2AFY**	0
**HDAC1**	27
**HIP1**	2
**HNRNPD**	4
**HNRNPK**	7
**HNRNPUL2**	0
**HSP90AB1**	7
**HSPG2**	3
**IRS1**	13
**ITGAV**	6
**ITGB1**	11
**ITGB3**	10
**ITGB5**	3
**KIRREL**	1
**KTN1**	2
**L1CAM**	4
**LCK**	21
**LEF1**	5
**LYN**	15
**MAGOH**	0
**MAPK3**	23
**MAVS**	2
**MDM2**	14
**MEF2A**	2
**MPRIP**	1
**MYB**	6
**MYBBP1A**	4
**MYC**	12
**MYH10**	2
**MYH9**	5
**MYLK**	6
**MYO1C**	0
**MYOF**	0
**MYOZ2**	3
**NCL**	10
**NCOR1**	7
**NDRG1**	8
**NDUFS1**	1
**NEDD9**	14
**NID1**	5
**NID2**	2
**NPHP1**	3
**NPM1**	4
**NR3C1**	12
**PABPC1**	5

**SVIL**	4
**SYK**	17
**SYNJ1**	7
**SYNM**	0
**TARDBP**	0
**TGFB1I1**	8
**THRAP3**	0
**TJP1**	12
**TLN1**	9
**TMEM30A**	0
**TOP1**	4
**TRABD**	0
**TRIP6**	4
**UBTF**	4
**UFSP2**	0
**UTP18**	0
**UXS1**	1
**VCL**	12
**VIM**	10
**YBX1**	6
**ZFPL1**	0

Supplementary Table 5. Directory of antibodies used

Target	Company	Catalog No	Application	Dilution
**Actinin-4**	Abcam	ab59468	IF	1:100
**Cleaved Caspase 3**	Cell Signaling	9664S	WB/IF	1:1,000/1:100
**p-Cofilin**	Cell Signaling	3313S	WB	1:1,000
**Cofilin**	Cell Signaling	5175S	WB	1:1,000
**GAPDH**	Sigma	G8795	WB	1:5,000
**p-LIMK**	Cell Signaling	3841S	WB/IF	1:1,000/1:100
**LIMK**	Cell Signaling	3842S	WB	1:1,000
**p-MAPK**	Cell Signaling	9216S	WB	1:1,000
**MAPK**	Cell Signaling	9106S	WB	1:1,000
**p-PAK**	Cell Signaling	2601S	WB	1:1,000
**PAK**	Cell Signaling	2604S	WB	1:1,000
**Paxillin**	Invitrogen	AHO0492	IF	1:200
**p-Src**	Cell Signaling	2101S	WB	1:1,000
**Src**	Cell Signaling	2123S	WB	1:1,000
**Synaptopodin**	Progen	65294	IF	1:50
**p-tyrosine**	Cell Signaling	9411S	IP	1:100
**WT1**	Abcam	ab89901	IF	1:50
**YAP**	Cell Signaling	4912S	IF	1:100

* WB = western blot, IF = immunofluorescence, IP = immunoprecipitation
