# Supplementary material for: Disruption of podocyte cytoskeletal biomechanics by dasatinib leads to nephrotoxicity
Source: Nat Commun. 2019 May 3;10:2061. doi: 10.1038/s41467-019-09936-x (PMC6499885; doi:10.1038/s41467-019-09936-x)
Supplement: Supplementary file 2 — Description of Additional Supplementary Files [file 41467_2019_9936_MOESM2_ESM.docx]

**Description of Supplementary Files**

**File Name:** Supplementary Data 1.

**Description:** Zip file containing adverse drug reaction analysis including the annotated script and the timestamped source data.
