# Supplementary material for: Disruption of podocyte cytoskeletal biomechanics by dasatinib leads to nephrotoxicity
Source: Nat Commun. 2019 May 3;10:2061. doi: 10.1038/s41467-019-09936-x (PMC6499885; doi:10.1038/s41467-019-09936-x)
Supplement: Supplementary file 4 — Reporting Summary [file 41467_2019_9936_MOESM4_ESM.pdf]

nature research

Corresponding author(s): Azeloglu, EU

Last updated by author(s): Apr 1, 2019

Reporting Summary

Nature Research wishes to improve the reproducibility of the work that we publish. This form provides structure for consistency and transparency in reporting. For further information on Nature Research policies, see Authors & Referees and the Editorial Policy Checklist.

Statistics

For all statistical analyses, confirm that the following items are present in the figure legend, table legend, main text, or Methods section.

n/a	Confirmed	
☐	☒	The exact sample size (*n*) for each experimental group/condition, given as a discrete number and unit of measurement
☐	☒	A statement on whether measurements were taken from distinct samples or whether the same sample was measured repeatedly
☐	☒	The statistical test(s) used AND whether they are one- or two-sided<br>*Only common tests should be described solely by name; describe more complex techniques in the Methods section.*
☐	☒	A description of all covariates tested
☐	☒	A description of any assumptions or corrections, such as tests of normality and adjustment for multiple comparisons
☐	☒	A full description of the statistical parameters including central tendency (e.g. means) or other basic estimates (e.g. regression coefficient) AND variation (e.g. standard deviation) or associated estimates of uncertainty (e.g. confidence intervals)
☐	☒	For null hypothesis testing, the test statistic (e.g. *F*, *t*, *r*) with confidence intervals, effect sizes, degrees of freedom and *P* value noted<br>*Give P values as exact values whenever suitable.*
☐	☒	For Bayesian analysis, information on the choice of priors and Markov chain Monte Carlo settings
☐	☒	For hierarchical and complex designs, identification of the appropriate level for tests and full reporting of outcomes
☐	☒	Estimates of effect sizes (e.g. Cohen's *d*, Pearson's *r*), indicating how they were calculated

Our web collection on statistics for biologists contains articles on many of the points above.

Software and code

Policy information about availability of computer code

Data collection	Custom CellProfiler, Matlab, and JavaScript codes were used for image processing. All custom code used in the manuscript is voluntarily made available in their entirety at the Azeloglu Lab GitHub page [https://github.com/AzelogluLab].
Data analysis	Custom Matlab codes were used for analysis of high-content data. All custom code used in the manuscript is voluntarily made available in their entirety at the Azeloglu Lab GitHub page [https://github.com/AzelogluLab].

For manuscripts utilizing custom algorithms or software that are central to the research but not yet described in published literature, software must be made available to editors/reviewers. We strongly encourage code deposition in a community repository (e.g. GitHub). See the Nature Research guidelines for submitting code & software for further information.

Data

Policy information about availability of data

All manuscripts must include a data availability statement. This statement should provide the following information, where applicable:

- Accession codes, unique identifiers, or web links for publicly available datasets
- A list of figures that have associated raw data
- A description of any restrictions on data availability

All data supporting the findings of this study are available from the corresponding author upon reasonable request. Raw mass spectrometry proteomics data have been deposited to the ProteomeXchange Consortium via the PRIDE partner repository with the dataset identifier PXD011761 and [https://www.ebi.ac.uk/pride/archive/projects/PXD011761].

Field-specific reporting

Please select the one below that is the best fit for your research. If you are not sure, read the appropriate sections before making your selection.

☒ Life sciences ☐ Behavioural & social sciences ☐ Ecological, evolutionary & environmental sciences

For a reference copy of the document with all sections, see nature.com/documents/nr-reporting-summary-flat.pdf

Life sciences study design

All studies must disclose on these points even when the disclosure is negative.

Sample size	For animal experiments, samples sizes were chosen based on prior experience and effect size estimates for normally distributed populations based on clinically derived relative odds ratios.
Data exclusions	No data was excluded.
Replication	Each experiment was repeated three times with at least three biological replicates, except for phosphoproteomics, which was repeated twice with three biological replicates.
Randomization	Littermates for drug vs. vehicle gavage groups were allocated randomly accounting for weight and sex.
Blinding	All quantitative histopathological sample preparation, imaging and analyses were performed by experts blinded at all levels. Only after complete data analysis the crypto was unveiled. No further reanalysis was performed.

Reporting for specific materials, systems and methods

We require information from authors about some types of materials, experimental systems and methods used in many studies. Here, indicate whether each material, system or method listed is relevant to your study. If you are not sure if a list item applies to your research, read the appropriate section before selecting a response.

Materials & experimental systems

n/a	Involved in the study	
☐	☒	Antibodies
☐	☒	Eukaryotic cell lines
☒	☐	Palaeontology
☐	☒	Animals and other organisms
☒	☐	Human research participants
☒	☐	Clinical data

Methods

n/a	Involved in the study	
☒	☐	ChIP-seq
☒	☐	Flow cytometry
☒	☐	MRI-based neuroimaging

Antibodies

Antibodies used	Actinin-4, Abcam (ab59468)<br>Cleaved Caspase 3, Cell Signaling (9664S)<br>p-Cofilin, Cell Signaling (3313S)<br>Cofilin, Cell Signaling (5175S)<br>GAPDH, Sigma (G8795<br>p-LIMK, Cell Signaling (3841S)<br>LIMK, Cell Signaling (3842S)<br>p-MAPK, Cell Signaling (9216S)<br>MAPK, Cell Signaling (9106S)<br>p-PAK, Cell Signaling (2601S)<br>PAK, Cell Signaling (2604S)<br>Paxillin, Invitrogen (AHO0492)<br>p-Src, Cell Signaling (2101S)<br>Src, Cell Signaling (2123S)<br>Synaptopodin, Progen (65294)<br>p-tyrosine, Cell Signaling (9411S)<br>WT1, Abcam (ab89901)<br>YAP, Cell Signaling (4912S)
Validation	All antibodies were tested on western blots to show bands at the correct size and cross-referenced to manufacturer data (uncropped blots are shown in Supplementary Figure 20). We also test all glomerular antibodies on frozen mouse/human tissue with immunofluorescence for spatial specificity. All phospho antibodies were validated through additional incubation of lysate

	with lambda phophatase. See Cell Signaling Technology website for additional validation performed by the manufacturer.

Eukaryotic cell lines

Policy information about cell lines

Cell line source(s)	Immortalized mouse podocytes were a gift from Peter Mundel (49). Immortalized mouse tubular epithelial cells were a gift from Luca Gusella (50).
Authentication	Podocytes stained positive for synaptopodin-crosslinked actin fibers. Tubular cells were tested to stain positive for AQP2 passages 1-12.
Mycoplasma contamination	All lines tested negative for mycoplasma using the Sigma Look-Out PCR Detection Kit. We test all our cell lines quarterly for mycoplasma.
Commonly misidentified lines (See ICLAC register)	N/A

Animals and other organisms

Policy information about studies involving animals; ARRIVE guidelines recommended for reporting animal research

Laboratory animals	129S1/SvImJ and MRL/MpJ-Faslpr mice both from Jackson Labs. All animals were 8-week old of equal distribution of both sexes.
Wild animals	N/A
Field-collected samples	N/A
Ethics oversight	All animal studies were approved and overseen by the Institute for Animal Care and Use Committee at Icahn School of Medicine at Mount Sinai. Mount Sinai animal facilities are AAALAC certified for ethical and humane treatment of animals.

Note that full information on the approval of the study protocol must also be provided in the manuscript.
